# *Aspergillus fumigatus* Elongator complex subunit 3 affects hyphal growth, adhesion and virulence through wobble uridine tRNA modification

**DOI:** 10.1371/journal.ppat.1010976

**Published:** 2022-11-14

**Authors:** Yuanwei Zhang, Yamei Wang, Jialu Fan, Guoxing Zhu, Ling Lu

**Affiliations:** Jiangsu Key Laboratory for Microbes and Functional Genomics, Jiangsu Engineering and Technology Research Center for Microbiology; College of Life Sciences, Nanjing Normal University, Nanjing, China; University of Melbourne, AUSTRALIA

## Abstract

The eukaryotic multisubunit Elongator complex has been shown to perform multiple functions in transcriptional elongation, histone acetylation and tRNA modification. However, the Elongator complex plays different roles in different organisms, and the underlying mechanisms remain unexplored. Moreover, the biological functions of the Elongator complex in human fungal pathogens remain unknown. In this study, we verified that the Elongator complex of the opportunistic fungal pathogen *Aspergillus fumigatus* consists of six subunits (Elp1-6), and the loss of any subunit results in similarly defective colony phenotypes with impaired hyphal growth and reduced conidiation. The catalytic subunit-Elp3 of the Elongator complex includes a S-adenosyl methionine binding (rSAM) domain and a lysine acetyltransferase (KAT) domain, and it plays key roles in the hyphal growth, biofilm-associated exopolysaccharide galactosaminogalactan (GAG) production, adhesion and virulence of *A*. *fumigatus*; however, Elp3 does not affect H3K14 acetylation levels *in vivo*. LC–MS/MS chromatograms revealed that loss of Elp3 abolished the 5-methoxycarbonylmethyl-2-thiouridine (mcm^5^s^2^U) modification of tRNA wobble uridine (U_34_), and the overexpression of tRNA^Gln^_UUG_ and tRNA^Glu^_UUC_, which normally harbor mcm^5^s^2^U modifications, mainly rescues the defects of the Δ*elp3* mutant, suggesting that tRNA modification rather than lysine acetyltransferase is responsible for the primary function of Elp3 in *A*. *fumigatus*. Strikingly, global proteomic comparison analyses showed significantly upregulated expression of genes related to amino acid metabolism in the Δ*elp3* mutant strain compared to the wild-type strain. Western blotting showed that deletion of *elp3* resulted in overexpression of the amino acid starvation-responsive transcription factor CpcA, and deletion of CpcA markedly reversed the defective phenotypes of the Δ*elp3* mutant, including attenuated virulence. Therefore, the findings of this study demonstrate that *A*. *fumigatus* Elp3 functions as a tRNA-modifying enzyme in the regulation of growth, GAG production, adhesion and virulence by maintaining intracellular amino acid homeostasis. More broadly, our study highlights the importance of U_34_ tRNA modification in regulating cellular metabolic states and virulence traits of fungal pathogens.

## Author summary

The opportunistic human pathogenic fungus *Aspergillus fumigatus* is able to sense nutrients in the environment to regulate growth and development, thus determining fungal adherence and virulence. Understanding the virulence traits of *A*. *fumigatus* provides insight and a molecular basis for the identification of novel drug targets. However, little is known about whether posttranscriptional modification affects nutrient sensing, fungal development and virulence in *A*. *fumigatus*. In this study, we demonstrate that the Elongator complex regulates hyphal development, conidiation, GAG production, adhesion and virulence through U_34_ tRNA modification by sensing intracellular amino acid levels and maintaining their homeostasis. Thus, the findings of this study demonstrate an important link between tRNA modification and amino acid sensing, which are involved in hyphal growth, GAG production, adhesion and virulence in human pathogenic fungi.

## Introduction

*Aspergillus fumigatus* is a well-known opportunistic pathogen that causes a wide variety of respiratory diseases, including aspergilloma, allergic bronchopulmonary aspergillosis (ABPA) and invasive aspergillosis (IA), and this fungus poses a major threat to immunocompromised individuals in clinical settings [1]. The limited repertoire of antifungal drug classes, the emergence of antifungal multidrug resistance and the toxicity caused by drugs negatively affect clinical outcomes and result in high rates of mortality and morbidity worldwide [2,3].

*A*. *fumigatus*, a successful human pathogen, has evolved a diverse range of sophisticated strategies to adapt to environmental constraints and to survive within and colonize the host; these strategies include its intrinsic thermotolerance, efficient dispersion in air and ability to adhere and form a biofilm [4]. These evolutionary strategies are precisely controlled at the transcriptional and translational levels. In terms of optimizing protein translation, transfer RNAs (tRNAs), which are key molecules involved in mRNA decoding during translation, undergo a multitude of posttranscriptional modifications that have profound impacts on protein synthesis [5]. The wobble base at position 34 (U_34_) in the anticodon stem loop (ASL) is the hotspot for tRNA modification [6]. Modifications in this region are important for optimizing translation elongation as they facilitate chemical interactions between the ASL and its codons during the ribosomal decoding process. Consistent with this finding, the loss of U_34_ modifications results in various translational defects, including reduced ribosomal A-site binding and decreased ribosomal translocation speed during translational elongation [7].

mcm^5^U (5-methoxycarbonylmethyluridine), mcm^5^s^2^U (5-methoxycarbonylmethyl-2-thiouridine) and ncm^5^U (5-carbamoylmethyluridine) are the most well understood U_34_ modifications and are catalyzed by a series of tRNA-modifying enzymes [8]. Among these tRNA-modifying enzymes, the Elongator complex has been shown to be responsible for introducing a carboxymethyl group to the C5-position of wobble uridine (cm^5^U) prior to its subsequent conversion to mcm^5^U, ncm^5^U or mcm^5^s^2^U by other downstream enzymes in the tRNA U_34_-modifying cascade [9]. The Elongator complex was initially identified as an elongating RNA polymerase II (RNAPII)-interacting protein in *Saccharomyces cerevisiae* [10], and it consists of two copies of six highly conserved subunits (Elp1–Elp6), with Elp1–3 forming the core subcomplex and Elp4–6 forming the accessory subcomplex [11]. Mutations and deficiencies in the subunits of the human Elongator complex are closely related to neurological disorders and cancers [12,13]. Loss of any of the six genes that encode the Elongator subunits in yeast results in similar phenotypes [14], such as increased sensitivity to salt, caffeine, temperature and 6-azouracil, indicating that the integrity of the Elongator complex is required for its full function.

Elp3 is a catalytic subunit of the Elongator complex, and it harbors an N-terminal radical S-adenosyl methionine domain (rSAM) and a C-terminal lysine acetyltransferase (KAT) domain [15]. The rSAM domain contains an iron-sulfur (Fe-S) cluster that can methylate tRNAs via its reductive SAM cleavage activity, and the KAT domain is very similar to the GCN5-related N-acetyltransferase (GNAT) family of lysine acetyltransferases, which acetylate lysine via their acetyl-CoA hydrolysis activity. Early findings showed that Elp3 is involved in the regulation of gene expression via histone acetylation, as evidenced by the reduced acetylation of lysine 14 of histone H3 (H3K14) and lysine 8 of histone H4 (H4K8) in yeast, plants and humans harboring the *elp3* null mutant [16–18]. In addition to histone proteins, Elp3 has been shown to be capable of acetylating ɑ-tubulin in postmitotic neurons, connexin-43 in the cerebral cortex and bruchpilot at the neuromuscular junction [19–21]. Recently, accumulating evidence suggests that Elp3 mainly performs its function through the modification of the tRNA wobble base uridine U_34_, and this modification promotes the decoding efficiency of the specific codons GAA, AAA and CAA [22]. *In vitro* reconstitution analysis and resolution of the crystal structure of Elp3 from the archaea *Methanocaldococcus infernus* revealed that both the rSAM and KAT domains are required for tRNA modification [23]. The iron-sulfur (Fe-S) cluster located in the rSAM domain is required to recruit and cleave SAM to generate a 5-deoxyadenosyl radical (5’-dA). The KAT domain is essential for acetyl-CoA hydrolysis, which is dependent on tRNA binding. The acetyl radical that is formed by the acetyl-CoA and 5’-dA generated by the two domains is added to the C5 position of the uridine base at position 34 of a tRNA [15]. The function of Elp3 in tRNA modification was further supported by results that showed that increased levels of the tRNA species tRNA^Gln^_UUG_, tRNA^Glu^_UUC_ and tRNA^Lys^_UUU_ suppressed the defects observed in the *elp3*-deficient mutant [24], including the reduced level of histone acetylation.

Despite growing evidence that shows the critical functions of the Elongator complex in eukaryotes, its biological role in the human fungal pathogen *A*. *fumigatus* and the underlying molecular mechanism remain unclear. In the present study, we showed that the catalytic subunit of the Elongator complex Elp3 regulates growth, biofilm-associated GAG production, adhesion, and virulence by functioning as a tRNA-modifying enzyme to maintain intracellular amino acid homeostasis. Our findings emphasize the critical role of tRNA modification in altering cellular metabolic states and fungal pathogen virulence traits.

## Results

### Lack of Elp3 impairs the hyphal growth and conidiation of *A*. *fumigatus*

**Fig 1:**
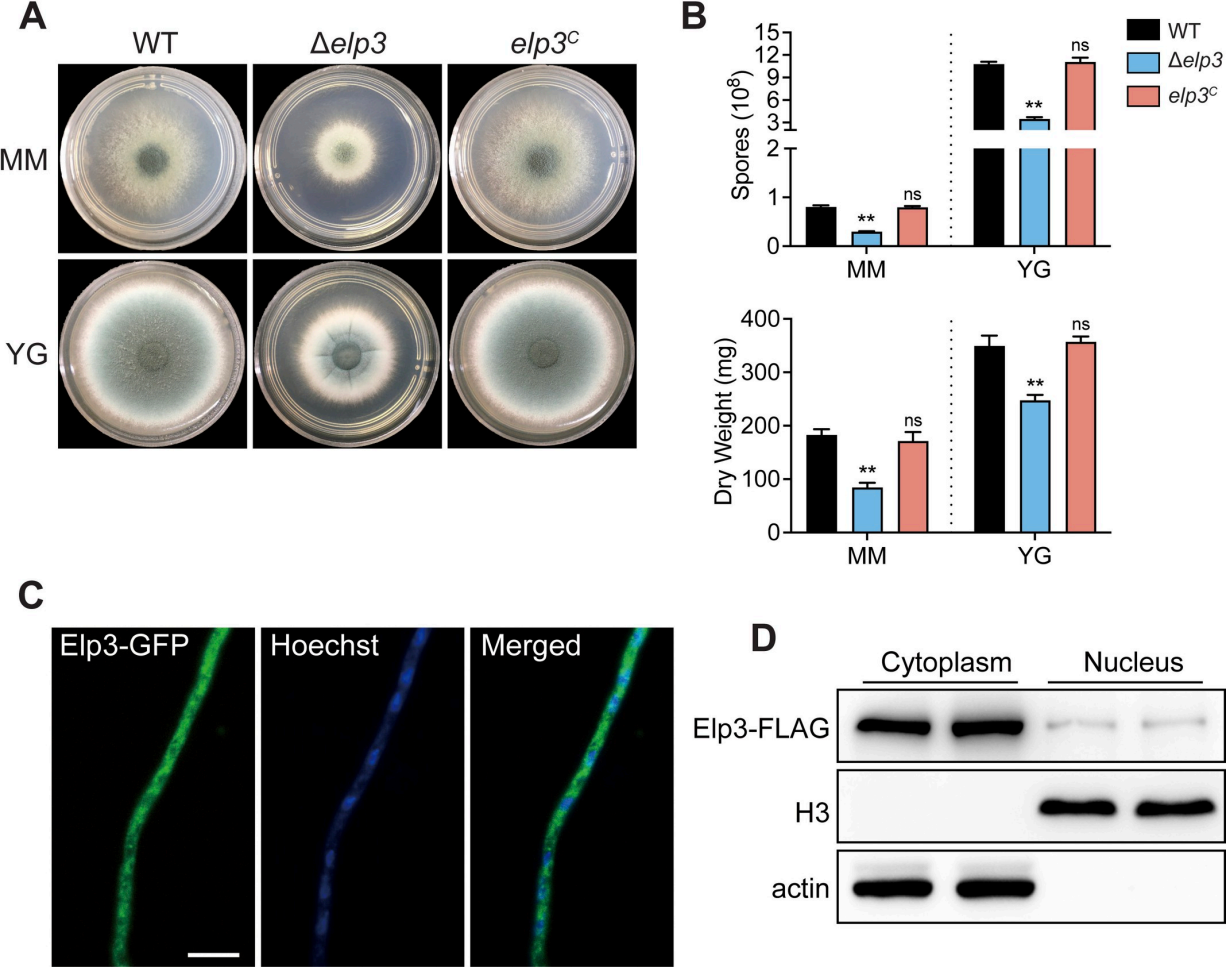
Elp3 is important for hyphal growth and conidiation in *A*. *fumigatus*. (A) Growth phenotypes of the wild-type, Δ*elp3* and complementation strains on solid and liquid minimal medium (MM) and complete YG medium at 37°C for 48 h. (B) Quantitative examination of spore production and biomass of the indicated strains. The data are presented as the mean ± SEM (standard error of the mean) of three independent experiments. Statistical analysis was performed using one-tailed, unpaired *t* tests. **p < 0.01; ns, not significant. (C) Localization of Elp3-GFP. Nuclei were stained with Hoechst 33258 stain. Scale bar = 10 μm. (D) Western blotting analysis of the distribution of Elp3 by subcellular fractionation. Actin and histone H3 were used as loading controls for the cytoplasmic and nuclear fractions, respectively.

To identify the putative Elp3 ortholog in *A*. *fumigatus*, a BLASTp search was performed against the *A*. *fumigatus* A1163 genome database using *S*. *cerevisiae* Elp3 as the query, and a single putative ortholog of the histone acetyltransferase Elp3 (AFUB_053700, NCBI accession: EDP51364.1) was identified. The full open reading frame (ORF) of *elp3* in *A*. *fumigatus* consists of 1725 bp with no introns and is predicted to encode a protein that is 575 amino acids in length. Domain architecture analysis revealed that Elp3 from *A*. *fumigatus* contains a radical S-adenosylmethionine (rSAM) domain and a histone acetyltransferase (HAT) domain (S1A Fig). A phylogenetic tree showed that Elp3 is highly conserved among fungi, humans, mice and plants, with more than 70% sequence identity (S1B Fig). To investigate the biological functions of Elp3 in *A*. *fumigatus*, a Δ*elp3*-null mutant and complemented strains were generated and verified by diagnostic PCR analysis (S2 Fig). As shown in Fig 1A, the Δ*elp3* mutant strain exhibited significantly reduced conidiation and hyphal growth on solid minimal medium (MM) and rich YG (yeast extract glucose) medium Compared to the wild-type (WT) and complemented strains. Quantitative analyses of conidia formation on solid medium showed that the number of conidia formed by the Δ*elp3* mutant on MM and YG media was significantly decreased compared to that formed by the WT and complemented strains, respectively (Fig 1B). Moreover, consistent with the decreased hyphal growth on solid medium, the Δ*elp3* mutant showed a significantly reduced biomass when submerged liquid MM and YG medium (Fig 1B). These results suggested that Elp3 is necessary for both hyphal growth and conidiation in *A*. *fumigatus*. To assess the localization of Elp3 in *A*. *fumigatus*, we labeled the C-terminus of Elp3 with green fluorescent protein (GFP) and expressed this fusion protein under the control of its native promoter. The Elp3-GFP strain exhibited an identical phenotype to the wild-type strain on solid media (S3 Fig), indicating that the Elp3-GFP fusion protein was fully functional. Fluorescence microscopy analysis revealed that the green fluorescence of the Elp3-GFP fusion protein was distributed throughout hyphal cells and overlapped with the blue fluorescence of the Hoechst nuclear stain (Fig 1C), suggesting that Elp3 localizes to the cytoplasm and nucleus. To confirm this finding, we also labeled Elp3 with a FLAG tag and expressed this fusion protein under the control of its native promoter; then, we assessed the localization of this fusion protein by subcellular fractionation and western blotting analysis. Consistent with the observations in the fluorescence microscopy images, subcellular fractionation revealed that a significant amount of Elp3-FLAG was present in the cytoplasm and a small fraction of Elp3 was present in the nucleus (Fig 1D).

### Loss of any elongator complex subunit results in similar defective phenotypes

**Fig 2:**
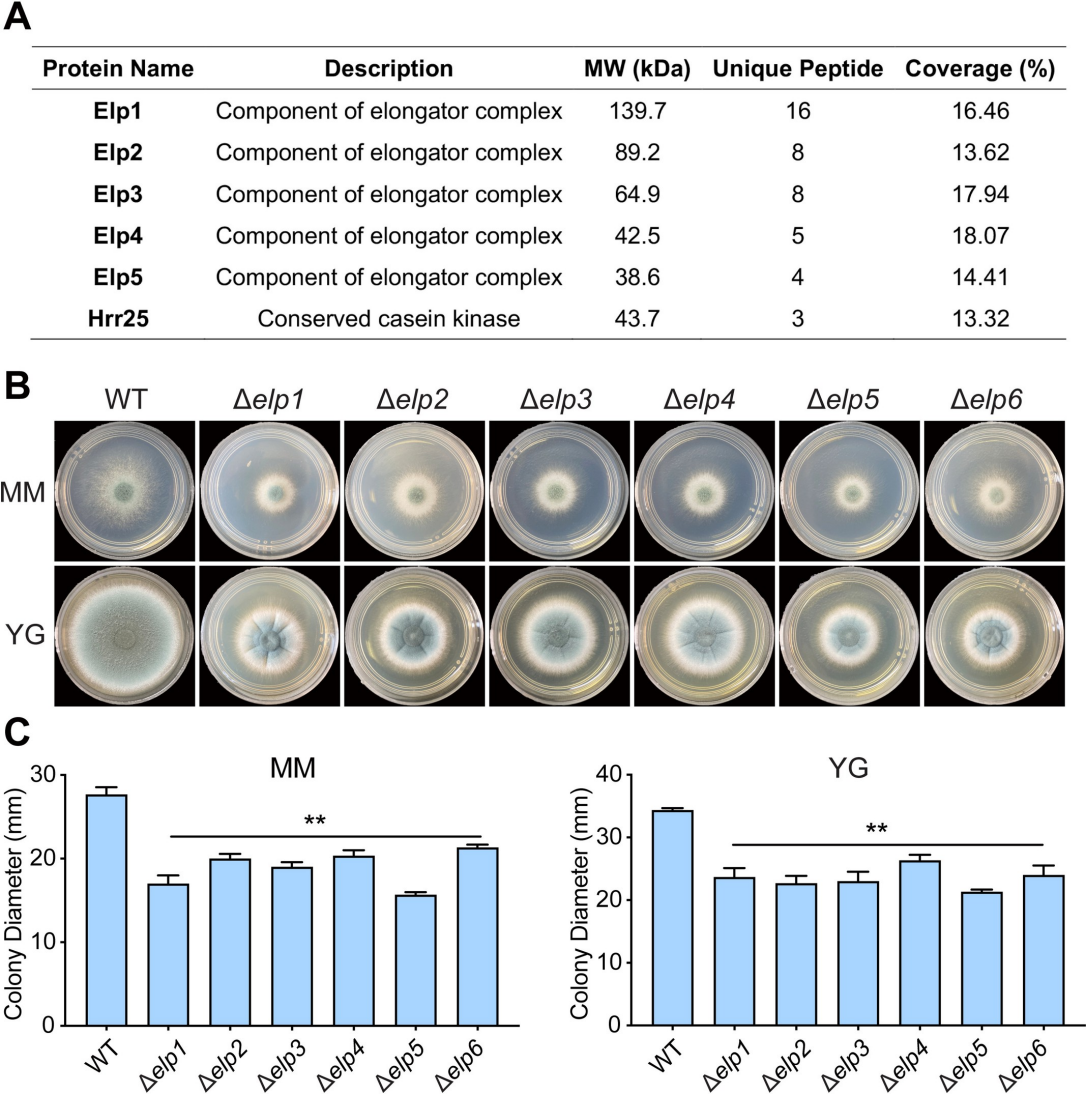
Disruption of each subunit of the Elongator complex leads to similar phenotypic defects. (A) Selected putative Elp3-interacting proteins identified by the FLAG pull-down assay. (B) Growth phenotypes of six Elongator complex subunit-null mutants on solid minimal medium (MM) and complete YG medium at 37°C for 48 h. (C) Quantitative examination of the diameters of the colonies formed by the indicated strains. The data are presented as the mean ± SEM (standard error of the mean) of three independent experiments. Statistical analysis was performed using one-way ANOVA with multiple comparisons tests. **p < 0.01; ns, not significant.

Given that Elp3 is a main catalytic subunit of the elongator complex, which comprises six subunits (Elp1, Elp2, Elp3, Elp4, Elp5, and Elp6) in *S*. *cerevisiae*, we wondered whether Elp3 interacts with other subunits to form a complex in *A*. *fumigatus*. To address this question, we performed coimmunoprecipitation (Co–IP) coupled with liquid chromatography tandem-mass spectrometry (LC–MS/MS) to identify Elp3-interacting proteins. C-terminus FLAG-tagged Elp3 strain under the control of its native promoter was constructed and the function of Elp3-FLAG fusion protein was verified by phenotypic analysis showing the identical phenotype to the parental wild-type strain (S3 Fig). FLAG-tagged Elp3 was isolated from total protein lysates by immunoprecipitation, and eluted proteins were digested with trypsin and characterized by LC–MS/MS. Specific Elp3-interacting proteins were identified by excluding proteins that were also coimmunoprecipitated from the negative control wild-type strain (S3 Table). As shown in Fig 2A, except for Elp6, all the putative subunits of the Elongator complex, including Elp1, Elp2, Elp4 and Elp5, were identified as potential Elp3-interacting proteins. These data suggested that Elp3 interacts with the other subunits to form the Elongator complex in *A*. *fumigatus*. In addition, the identified casein kinase Hrr25 was previously shown to interact with Elp3 and is required for the activity of the Elongator complex in budding yeast [25], further validating our results. Next, we inoculated the conidia of all six Elongator complex subunit-null mutants onto solid minimal media and rich media to examine the morphology of the colonies formed by each mutant. As shown in Fig 2B and 2C, phenotypic analysis revealed that deletion of the other subunits of the Elongator complex (Elp1, 2, 4, 5, and 6) resulted in defective phenotypes that were similar to that observed in the Δ*elp3* mutant, with reduced hyphal growth and conidiation. Taken together, these results suggested that each subunit of the Elongator complex is required for its biological functions in *A*. *fumigatus*.

### Elp3 modulates the expression of genes involved in carbohydrate, lipid and amino acid metabolism and GAG biosynthesis

**Fig 3:**
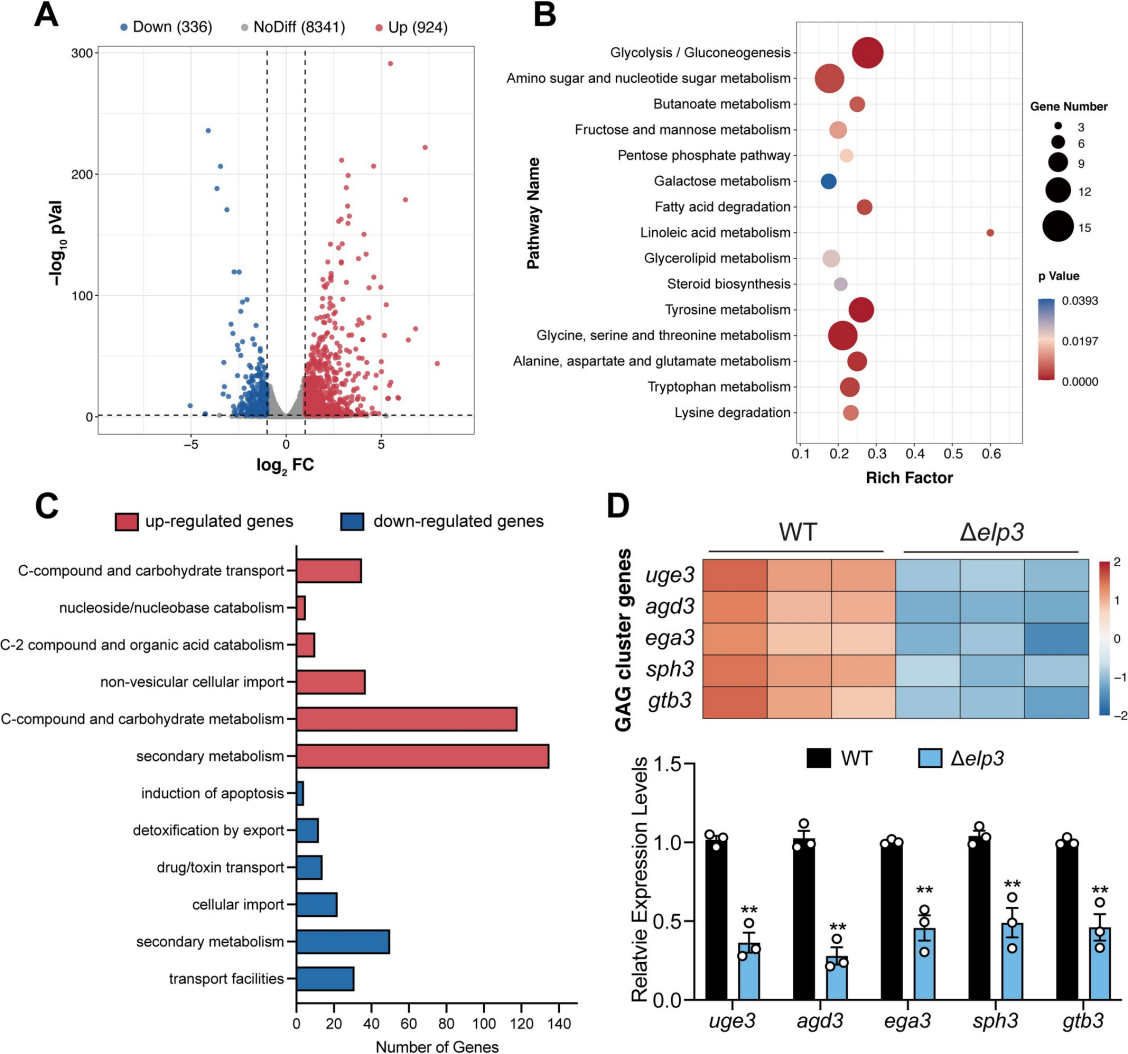
Loss of Elp3 alters the expression of genes involved in carbohydrate, lipid and amino acid metabolism and GAG biosynthesis. (A) Volcano plot showing the fold change and statistical significance of the differences in quantitative mRNA expression between the wild-type and Δ*elp3* strains grown at 37°C for 24 h. (B) KEGG pathway enrichment analysis of differentially expressed genes in the wild-type strain versus Δ*elp3* mutant strain. (C) Gene ontology analysis of significantly upregulated and downregulated genes in the Δ*elp3* mutant strain compared to the wild-type strain. (D) Heatmap of GAG cluster gene expression and quantitative real-time RT–PCR analysis of GAG cluster genes in the wild-type and Δ*elp3* strains. The mRNA levels were normalized to that of the reference gene *tubA*. The data are presented as the mean ± SEM (standard error of the mean) of three independent experiments. Statistical analysis was performed using one-tailed, unpaired *t* tests. **p < 0.01.

To elucidate the molecular mechanism underlying the defective phenotypes caused by the loss of Elp3, we measured global gene expression changes in the Δ*elp3* mutant strain compared to the wild-type strain by RNA-seq (S4 Table). The expression levels of 336 genes and 924 genes were downregulated and upregulated by at least twofold in the Δ*elp3* mutant compared to the wild-type strain, respectively (log_2_FC ≥ 1.0 and ≤ -1.0; *p* value < 0.05) (Fig 3A). KEGG pathway enrichment analysis of the differentially expressed genes (DEGs) revealed that the significantly enriched pathways were carbohydrate metabolism, lipid metabolism and amino acid metabolism (Fig 3B). FunCat (Functional Catalogue) enrichment analysis (https://elbe.hki-jena.de/fungifun) of the upregulated genes revealed enrichment of genes involved in secondary metabolism, C-compound and carbohydrate metabolism, C-compound and carbohydrate transport, and nonvesicular cellular import (Fig 3C). In contrast, FunCat functional categories enriched in the downregulated genes included transport facilities, secondary metabolism, cellular import, and drug/toxin transport (Fig 3C). Of note, among the differentially expressed genes dependent on Elp3, we noticed that the expression of a gene cluster in the exopolysaccharide galactosaminogalactan (GAG) biosynthesis pathway (*uge3*, *ega3*, *agd3*, *gtb3*, and *sph3*) [26], which is required for biofilm formation, was significantly downregulated, and these results were further verified by qRT–PCR analysis (Fig 3D). Taken together, these results indicated that Elp3 influences fungal carbohydrate, lipid and amino acid metabolism and GAG biosynthesis.

### Elp3 deficiency causes reduced GAG production, adhesion and attenuated virulence in *A*. *fumigatus*

**Fig 4:**
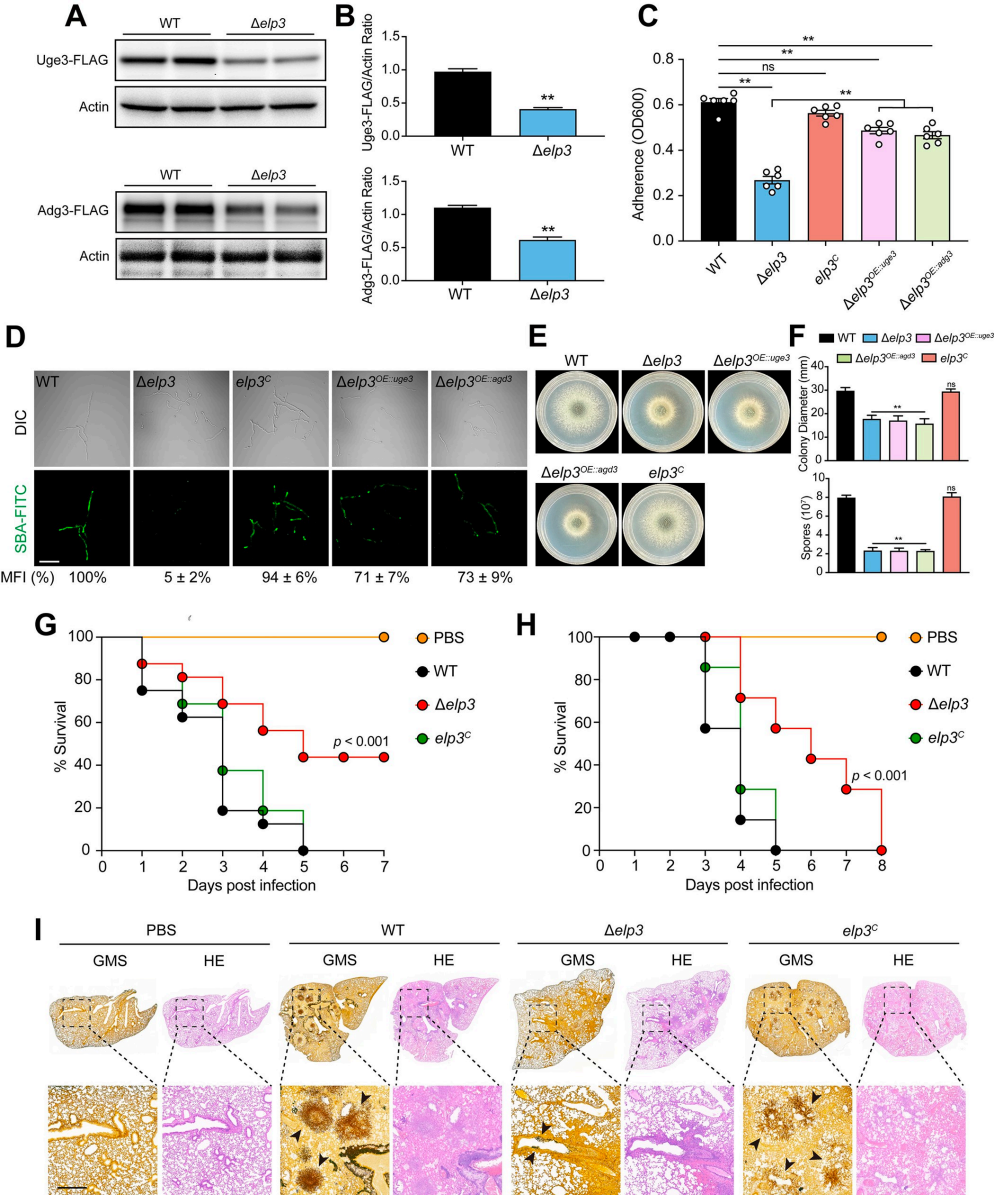
The *elp3* null mutant exhibits reduced GAG production, adhesion and attenuated virulence. (A) Western blots show the protein expression of Uge3 and Agd3 in the wild-type and Δ*elp3* strains. β-actin served as a loading control. (B) Quantified western blot signal intensity for Uge3-FLAG and Agd3-FLAG relative to actin in the wild-type and Δ*elp3* strains. The data are presented as the mean ± SEM (standard error of the mean) of three independent experiments. Statistical analysis was performed using one-tailed, unpaired *t* tests. **p < 0.01. (C) Quantification of adherence ability of the indicated strains by crystal violet staining. The data are presented as the mean ± SEM (standard error of the mean) of three independent experiments. Statistical analysis was performed using one-way ANOVA with multiple comparisons tests. **p < 0.01; ns, not significant. (D) GAG production of the indicated strains was visualized by soybean agglutinin (SBA) lectin staining. Scale bar = 10 μm. The mean fluorescence intensity (MFI) of SBA-FITC in the indicated strains was calculated and normalized to the wild-type (100%). (E) Growth phenotypes of Δ*elp3*^*OE*::*uge3*^ and Δ*elp3*^*OE*::*agd3*^ strains on solid minimal medium at 37°C for 48 h. (F) Quantitative examination of the diameters and conidiation of the indicated strains. The data are presented as the mean ± SEM (standard error of the mean) of three independent experiments. Statistical analysis was performed using one-way ANOVA with multiple comparisons tests. **p < 0.01; ns, not significant. (G and H) Survival curves of *G*. *mellonella* larvae (G) and mice (H) infected with the wild-type, Δ*elp3*, and complementation strains. PBS-injected larvae and mice were used as negative controls. Statistical differences between groups were determined using a log-rank test. (I) Histology of lungs harvested from mice infected with the indicated *A*. *fumigatus* strains. Lung sections were stained with hematoxylin and eosin (H&E) to visualize the host cells and Grocott’s methenamine silver (GMS) to visualize the fungal hyphae. The black arrow indicates the fungal mycelia.

To further confirm that the expression of GAG biosynthesis cluster genes was decreased in the Δ*elp3* mutant, we next sought to assess the expression patterns of these molecules at the protein level. We labeled Uge3 and Agd3 with a FLAG tag at the C-terminus and expressed these fusion proteins under the control of their native promoters in the wild-type and Δ*elp3* strains. Western blotting analysis showed that the protein expression of Uge3 and Agd3 was decreased in the Δ*elp3* mutant (Fig 4A and 4B). This is consistent with the reduced expression of the GAG cluster gene, which is required for GAG production and adhesion. Consistently, the Δ*elp3* mutant exhibited reduced adherence to polystyrene plates according to crystal violet staining (Fig 4C). In addition, the reduced GAG production in the Δ*elp3* mutant was further confirmed by GAG-specific soybean agglutinin lectin (SBA-FITC) staining; in this assay, the Δ*elp3* mutant strain displayed weaker fluorescence than the wild-type strain (Fig 4D). Notably, overexpression of *uge3* or *adg3* partially restored the GAG production and adhesion defects of the Δ*elp3* mutant, but not for the growth defects (Fig 4C–4F). These results confirmed that the impaired GAG production and adhesion ability of the Δ*elp3* mutant occurred due to the reduced expression of GAG cluster genes. Similar phenotypes were also observed in strains harboring point mutations in the Elp3 rSAM and KAT domains (S4 Fig). These results suggested that Elp3 plays an important role in GAG production and adhesion in *A*. *fumigatus*.

Given that deletion of *elp3* results in defective phenotypes that are relevant to pathogenesis, such as inhibited hyphal growth and GAG production, we wondered whether Elp3 affects fungal virulence in *A*. *fumigatus*. To this end, we compared the virulence potential of the wild-type, Δ*elp3*, and *elp3*^C^ complementary strains using the *G*. *mellonella* wax moth and murine models. *G*. *mellonella* larvae were injected with the conidia of the indicated strains and incubated at 37°C for 7 days. As shown in Fig 4G, the Δ*elp3* mutant had a higher rate of larval survival than the wild-type and complemented strains. The percentage of surviving larvae 7 days after infection with the Δ*elp3* mutant was approximately 40%, while at the end of the fifth day, all the larvae infected with the wild-type and complemented strains were dead. No mortality was observed in the PBS-injected group. In a murine model of invasive aspergillosis, survival curve analysis showed that the Δ*elp3* mutant strain was significantly less virulent than the wild-type and complemented strains (Fig 4H). At 5 days post-infection, all the mice infected with the wild-type and complemented strains were dead, whereas 60% of mice infected with the Δ*elp3* mutant were alive. Similarly, histopathological analysis showed that the lungs of the mice infected with the wild-type and complemented strains exhibited strong inflammatory responses, and these lungs were characterized by robust hyphal growth, and leukocyte infiltration into bronchovascular, and granuloma formation (Fig 4I). In contrast, no obvious hyphal cluster or inflammation was observed in the Δ*elp3*-infected and PBS-injected mice at the same time point. Collectively, these results suggested that a lack of Elp3 attenuates the virulence of *A*. *fumigatus* in the *G*. *mellonella* insect model and in murine models of invasive aspergillosis.

### The conserved tRNA modification-related residues in the rSAM and KAT domains of Elp3 contribute to hyphal growth and conidiation in *A*. *fumigatus*

**Fig 5:**
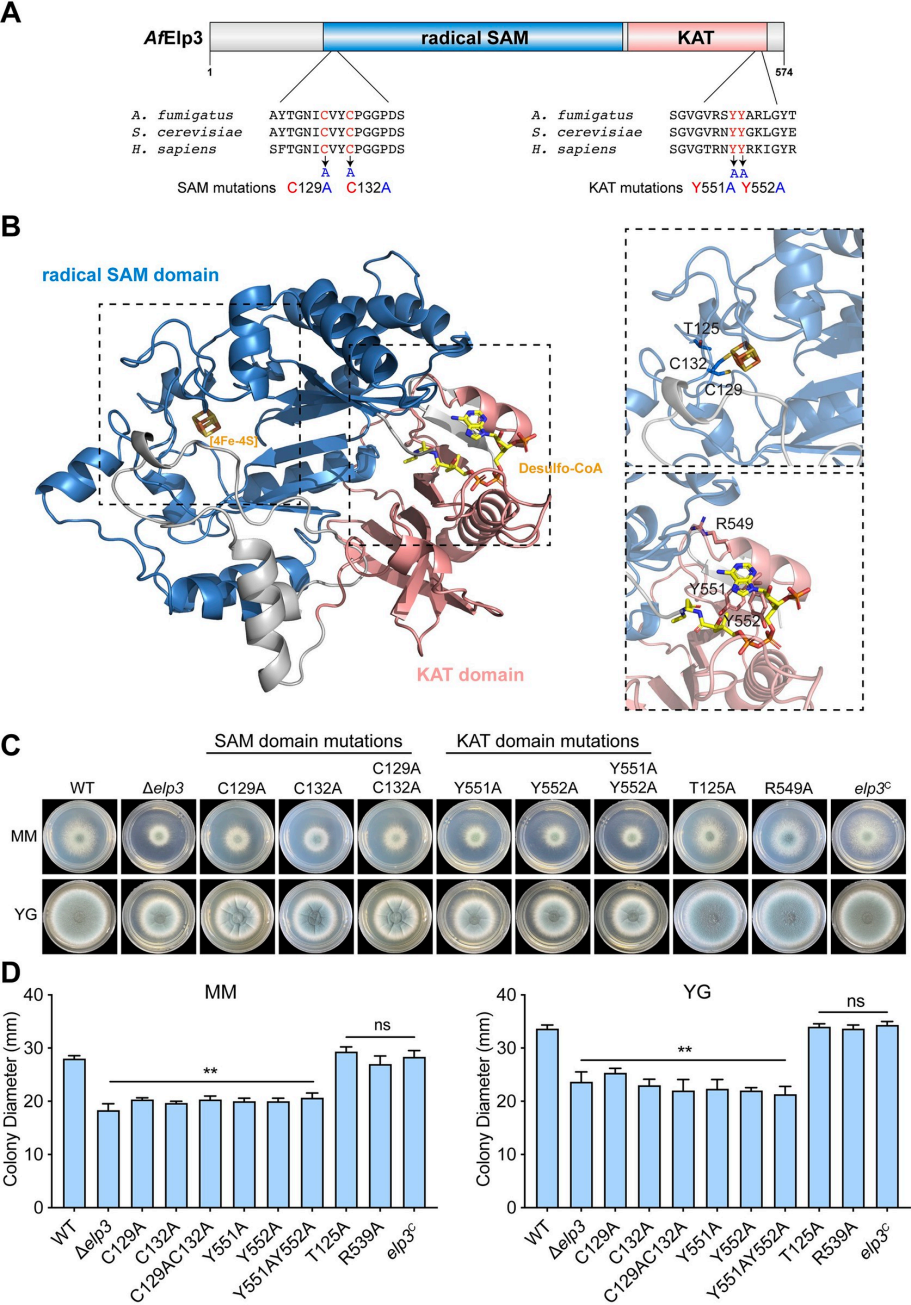
The conserved residues in the rSAM and KAT domains of Elp3 are required for hyphal growth and conidiation in *A*. *fumigatus*. (A) Schematic diagram of the *A*. *fumigatus* Elp3 protein. The blue box represents the radical-S-adenosyl methionine (rSAM) domain, and the pink box indicates the lysine acetyltransferase (KAT) domain. Cys129 and 132 were substituted with alanine, and Tyr551 and 552 were replaced by alanine. (B) Predicted crystal structure of *A*. *fumigatus* Elp3 bound to the [4Fe4S] cluster and desulfo-CoA. The rSAM and KAT domains are shown as blue and pink ribbons, respectively. Cysteine residues (C129 and C132) and threonine residues (T125) in the rSAM domain and tyrosine residues (Y551 and Y552) and arginine residues (R549) in the KAT domain are shown as sticks. (C) Colony morphology of the wild-type, Δ*elp3*, site-directed mutants, and complementation strains grown on solid minimum medium (MM) and complete YG medium at 37°C for 48 h. (D) Quantitative analysis of the diameter of the colonies formed by the indicated strains grown at 37°C for 48 h. The data are presented as the mean ± SEM (standard error of the mean) of three independent experiments. Statistical analysis was performed using one-way ANOVA with multiple comparisons tests. **p < 0.01; ns, not significant.

Elp3 contains a radical SAM and KAT domain, both of which are highly conserved among species (Fig 5A). To test whether the KAT and the radical SAM domains of Elp3 are required for the function of the Elongator complex in *A*. *fumigatus*, we used the SwissModel server to build the homology model of *A*. *fumigatus* Elp3 using *S*. *cerevisiae* Elp3 (PDB ID: 6QK7) as a template [11]. Structural superposition of *A*. *fumigatus* Elp3 with the structure of *Sc*Elp3 revealed that two conserved cysteine residues (C129 and C132) in the rSAM domain and two conserved tyrosine residues (Y551 and Y552) in the KAT domain are coordinated to the Fe-S cluster and desulfo-CoA, respectively (Fig 5B). The binding sites of the Fe-S cluster and desulfo-CoA were previously shown to be essential for the function of Elp3 in tRNA modification at U_34_ in yeast, and mutations of these sites completely abolished tRNA modification activity [27]. To determine whether these residues are required for the function of Elp3, we then constructed mutants in which two conserved cysteine residues were changed to alanine and two conserved tyrosine residues were changed to alanine by site-directed mutagenesis and the introduction of exogenously mutated genes into the Δ*elp3* strain. As controls, we mutated T125 (threonine) and R549 (arginine), which were not predicted binding sites, to alanine. qRT–PCR analysis showed that the expression levels of mutated genes were similar to those of the wild-type *elp3* gene, indicating that the transcription of the mutated genes was unaffected (S5 Fig). As shown in Fig 5C and 5D, the mutant strains carrying the single mutations C129A, C132A, Y551A, or Y552A or the double mutations C129AC132A or Y551AY552A displayed reduced radial growth and conidiation, which is similar to the phenotypes of the Δ*elp3* mutant strain. In comparison, the strains carrying the T125A and R549A mutations showed no detectable difference compared to the parental wild-type strain. Collectively, these results suggested that the conserved tRNA modification-related residues in the rSAM and KAT domains of Elp3 play a role in hyphal development and conidiation in *A*. *fumigatus*.

### Phenotypes of the *elp3* deletion mutant are associated with defects in tRNA modification

**Fig 6:**
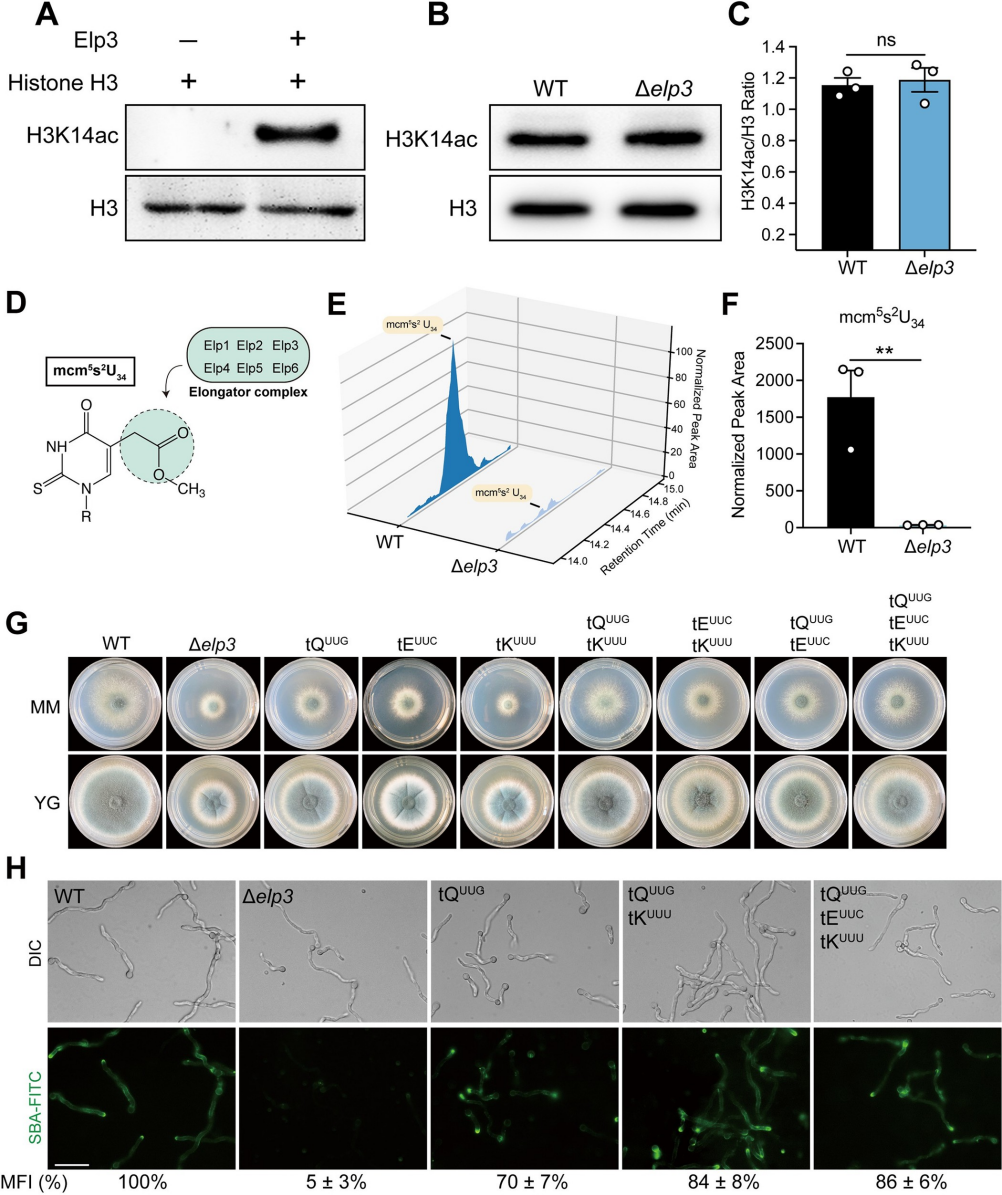
Defective phenotypes of the Δ*elp3* mutant are associated with tRNA modification defects. (A) *In vitro* histone acetyltransferase assays. Purified Elp3 was incubated with HeLa core histones as substrates. Acetylation levels were measured by western blotting using H3K14ac antibodies. (B) Western blotting analysis of acetylated H3K14 levels in the wild-type and Δ*elp3* strains. Total histone H3 served as a protein loading control. (C) Quantified western blot signal intensity of H3K14ac relative to H3 in the indicated strains. The data are presented as the mean ± SEM (standard error of the mean) of three independent experiments. Statistical analysis was performed using one-tailed, unpaired *t* tests. ns, not significant. (D and E) LC–MS/MS chromatograms of mcm^5^s^2^U modification of tRNA from the wild-type and Δ*elp3* strains. (F) Quantification of mcm^5^s^2^U wobble modification of tRNAs in the wild-type and Δ*elp3* strains. The peaks of modified nucleoside were extracted and normalized to the quantity of tRNA purified. The data are presented as the mean ± SEM (standard error of the mean) of three independent experiments. Statistical analysis was performed using one-tailed, unpaired *t* tests. **p < 0.01. (G) Colony morphology of the indicated strains grown on solid minimum medium (MM) and complete YG medium at 37°C for 48 h. (H) GAG production of the indicated strains was visualized by soybean agglutinin (SBA) lectin staining. Scale bar = 10 μm. The mean fluorescence intensity (MFI) of SBA-FITC in the indicated strains was calculated and normalized to the wild-type (100%).

A previous study showed that Elp3 performs its function via the acetylation of histone H3 lysine-14 residues (H3K14ac), and deletion of *elp3* leads to decreased H3K14ac levels [17]. To examine whether the Elp3 of *A*. *fumigatus* possesses lysine acetyltransferase (KAT) activity, we incubated the purified Elp3 protein with HeLa cell core histones H3–H4 as substrates and assessed the KAT activity *in vitro*. As shown in Fig 6A, the acetylation of H3K14 was clearly observed by western blotting analysis, suggesting that Elp3 possesses histone acetyltransferase activity *in vitro*. Next, we further investigated whether Elp3 could affect H3K14 acetylation *in vivo*. Total proteins were extracted from the wild-type and Δ*elp3* strains and then subjected to western blotting analysis. Unexpectedly, the acetylation levels of H3K14 in the Δ*elp3* mutant were comparable with those in the wild type (Fig 6B and 6C), suggesting that deletion of *elp3* did not affect H3K14 acetylation in *A*. *fumigatus in vivo*. These results led us to hypothesize that the defective phenotypes observed in the Δ*elp3* mutant might not correlate with H3K14 acetylation level. To test this hypothesis, we constructed the H3K14R mutant strain, in which K14 was substituted with arginine to mimic the unacetylated form of lysine. Compared to the Δ*elp3* mutant strain, the H3K14R mutant strain showed only slightly reduced hyphal growth (S6A Fig). More importantly, the mRNA levels of GAG cluster gene expression and GAG production were not affected in the H3K14R mutant strain (S6B and S6C Fig), thus confirming that H3K14 acetylation level is not related to the defective phenotypes of the Δ*elp3* mutant. In yeast, the Elongator complex was shown to be required for tRNA modification at the wobble uridine 34 (U_34_), for example, via the formation of the 5-methoxycarbonylmethyl-2-thiouridine modification (mcm^5^s^2^) side chain at the U_34_ site (Fig 6D) [27]. Overexpression of hypomodified tRNA reversed all the phenotypes observed in Elongator subunit-mutant yeast [24,27]. To investigate whether Elp3 is required for tRNA modification in *A*. *fumigatus*, we quantified the abundance of mcm^5^s^2^ modifications by LC–MS/MS analysis. As shown in Fig 6E and 6F, the formation of mcm^5^s^2^ was almost abolished in the Δ*elp3* mutant, indicating the critical role of Elp3 in tRNA modification. Moreover, similar to previous findings in yeast, the overexpression of the tRNAs tRNA^Gln^_UUG_, tRNA^Glu^_UUC_ and tRNA^Lys^_UUU_, alone or in combination, restored the defects in hyphal growth observed in the Δ*elp3* mutant to different extents, and tRNA^Gln^_UUG_ was the most efficient at suppressing these defects in *A*. *fumigatus* (Figs 6G and S7A). More importantly, the reduced expression of GAG cluster genes and defects in GAG production were also suppressed by the overexpression of tRNA, as shown by SBA-FITC staining (Figs 6H and S7B). Together, these results suggested that the phenotypes of the *elp3* deletion mutant are associated with defects in tRNA modification.

### Elp3 is involved in sensing amino acid availability, and disruption of the amino acid-regulating transcription factor CpcA rescues the defective phenotypes in Δ*elp3*

**Fig 7:**
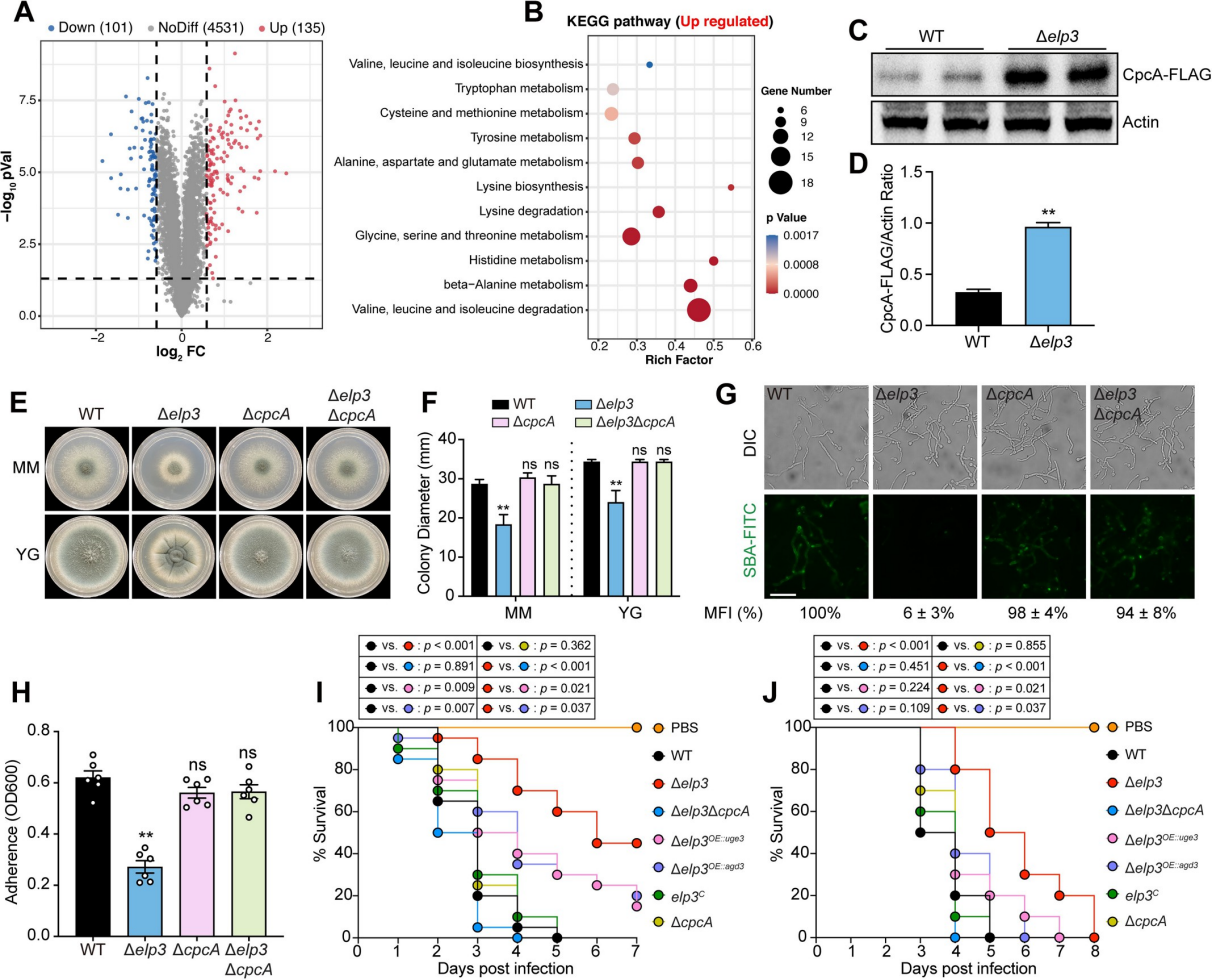
Deletion of CpcA restored the defects of the Δ*elp3* mutant, including reduced hyphal growth, GAG production, adhesion and virulence. (A) Volcano plot showing the fold change and statistical significance of the differences in quantitative protein expression between the wild-type and Δ*elp3* strains grown at 37°C for 24 h. (B) KEGG pathway enrichment analysis of upregulated proteins in the Δ*elp3* mutant strain compared to the wild-type strain. (C) Western blots of the protein expression of CpcA in the wild-type and Δ*elp3* strains. β-actin served as a loading control. (D) Quantified western blot signal intensity of CpcA-FLAG relative to actin in the indicated strains. The data are presented as the mean ± SEM (standard error of the mean) of three independent experiments. Statistical analysis was performed using one-tailed, unpaired *t* tests. **p < 0.01. (E) Morphologies of the colonies formed by wild-type, Δ*elp3*, Δ*cpcA* and Δ*elp3*Δ*cpcA* double mutants grown on solid minimum medium (MM) and complete YG medium at 37°C for 48 h. (F) Quantitative analysis of the diameters of the colonies formed by the indicated strains grown at 37°C for 48 h on solid minimum medium (MM) and complete YG medium. The data are presented as the mean ± SEM (standard error of the mean) of three independent experiments. Statistical analysis was performed using one-way ANOVA with multiple comparisons tests. **p < 0.01; ns, not significant. (G) GAG production of the indicated strains was visualized by soybean agglutinin (SBA) lectin staining. Scale bar = 10 μm. The mean fluorescence intensity (MFI) of SBA-FITC in the indicated strains was calculated and normalized to the wild-type (100%). (H) Quantification of adhesion ability by the indicated strains by crystal violet staining. The data are presented as the mean ± SEM (standard error of the mean) of three independent experiments. Statistical analysis was performed using one-tailed, unpaired *t* tests. **p < 0.01; ns, not significant. (I and J) Survival curves of *G*. *mellonella* larvae (I) and mice (J) infected with the indicated strains. PBS-injected larvae and mice were used as negative controls. Statistical differences between groups were determined using a log-rank test.

It has been proposed that the pleiotropic phenotypes of the *elp3* deletion mutant could be attributed to the reduced expression of specific proteins whose mRNA transcripts are enriched in CAA, GAA and AAA codons or to proteotoxic stress caused by global protein aggregation [28–30]. To investigate the underlying mechanism by which Elp3 regulates the hyphal growth, GAG production and adhesion of *A*. *fumigates*, we performed global proteomic analyses of the Δ*elp3* mutant compared with the wild-type strain (S5 Table). The expression of 101 proteins was significantly downregulated in the Δ*elp3* mutant (log_2_FC ≤ -0.58; *p* value < 0.05) (Fig 7A). These genes were not functionally enriched for any gene ontology categories according to FunCat analysis. Analysis of the codon usage within these downregulated genes showed that only a few proteins with unknown functions displayed a prominent codon bias of AAA, CAA, and GAA (S8 Fig). In addition, we did not observe significant upregulation of unfolded protein response-related gene expression in the Δ*elp3* mutant (S9 Fig), suggesting that global protein aggregation may not occur. These findings led us to speculate that other mechanisms might be involved in Elp3-dependent hyphal growth, GAG production and adhesion. Notably, we found that the upregulated genes were significantly enriched for amino acid metabolism (Fig 7B), such as valine, leucine and isoleucine biosynthesis, cysteine and methionine metabolism, and glycine, serine and threonine metabolism; this finding is consistent with a previous study showing the increased expression of genes related to amino acid biosynthetic in cells lacking U_34_ modification [31]. The mutant that lacked U_34_ modification exhibited an amino acid starvation state and overexpressed the amino acid starvation-responsive transcription factor Gcn4. Western blotting analysis showed that the expression of CpcA, the homolog of *S*. *cerevisiae* Gcn4 in *A*. *fumigatus*, was significantly increased in the Δ*elp3* mutant strain compared to the wild-type strain (Fig 7C and 7D), indicating that CpcA is overexpressed in the Δ*elp3* mutant strain. Next, we wondered whether CpcA overexpression contributes to the phenotypes of the Δ*elp3* strain. To test this hypothesis, we knocked out the *cpcA* gene in the Δ*elp3* mutant strain and examined the phenotypes of the double mutant strain. Strikingly, deletion of *cpcA* from the Δ*elp3* mutant completely rescued the impaired hyphal growth, conidiation, GAG production and adhesion as shown by SBA-FITC staining and crystal violet assay (Fig 7E–7H). Together, these results suggested that deletion of CpcA restored the defects of the Δ*elp3* mutant, including reduced hyphal growth, GAG production, adhesion and attenuated virulence. Next, we further asked whether attenuated virulence of the Δ*elp3* mutant in the insect and murine models was due to growth defects or reduced GAG production. To determine this, we compared the virulence of Δ*elp3*Δ*cpcA* double mutant, Δ*elp3*^*OE*::*uge3*^, Δ*elp3*^*OE*::*agd3*^ with the Δ*elp3* mutant. As shown in Fig 7I and 7J, deletion of *cpcA* was able to restore nearly full virulence of the Δ*elp3* mutant in both wax moth and murine models, whereas overexpression of *uge3* or *agd3* in the Δ*elp3* mutant partially restore virulence, suggesting that both growth defects and reduced GAG production contribute to the attenuated virulence in the Δ*elp3* mutant.

## Discussion

**Fig 8:**
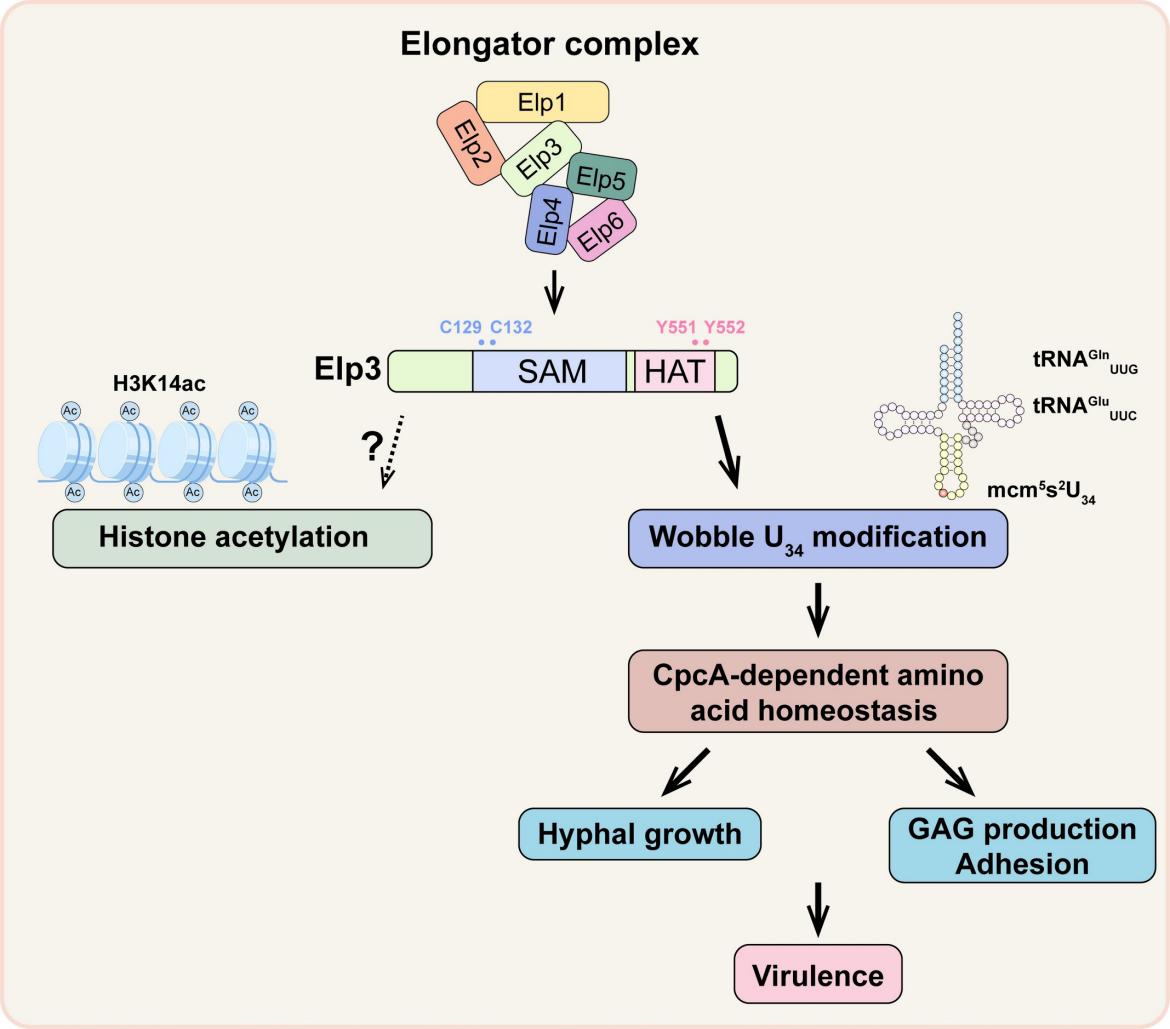
A proposed working model of the mechanism by which the Elongator complex mutant impairs hyphal growth, conidiation, GAG production, adhesion and virulence in *A*. *fumigatus*.

The Elongator complex, which is conserved from yeasts to humans, has been shown to be involved in a wide range of biological processes. In contrast to its highly conserved structure, the regulatory mechanisms of the Elongator complex are diverse and species specific [32]. In this study, we elucidated the previously uncharacterized functions of the catalytic subunit Elp3 of the Elongator complex in the human fungal pathogen *A*. *fumigatus*. Our results showed that each subunit of the Elongator complex is required for the full function of the complex and that Elp3 acts as a tRNA-modifying enzyme rather than a lysine acetyltransferase to mediate the hyphal growth, conidiation, GAG production, adhesion and virulence of *A*. *fumigatus*. Loss of Elp3 leads to the loss of mcm^5^s^2^ modification at the tRNA wobble base U_34_, followed by an amino acid starvation state accompanied by CpcA overexpression. As a result, the rewired cellular metabolism results in impaired hyphal growth, GAG production, adhesion and virulence in *A*. *fumigatus* in a tRNA modification-dependent manner (Fig 8).

The highly evolutionarily conserved structure of the Elongator complex implies that it performs critical biological functions in many species. As a catalytic subunit of the Elongator complex, Elp3 is present in all three domains of life, including all archaea and some clades of bacteria [23]. Disruption of Elp3 results in pleiotropic phenotypes, including inhibited growth and development and altered stress responses in plants and fungi [27,33–36]. Similarly, our phenotypic characterization revealed that loss of Elp3 leads to reduced hyphal growth and spore production in *A*. *fumigatus* (Fig 1), indicating the indispensable role of Elp3 in hyphal growth and conidiation. In yeast and *F*. *graminearum*, deletion of *elp3* causes hypersensitivity to oxidative stress [37,38], while the Δ*elp3* mutant strain of *A*. *fumigatus* showed resistance to H_2_O_2_ and menadione (S10 Fig). These results reflected the divergent role of Elp3 in stress responses among species. In addition, the FLAG-trap assay showed that Elp3 interacts with other subunits to form a complex in *A*. *fumigatus*, and more importantly, disruption of any single subunit of the Elongator complex results in similar defective phenotypes (Fig 2), which is consistent with the conclusions drawn from previous studies in yeast and plants showing that all six Elongator subunits are indispensable and form an integral complex to perform their proper functions.

In addition to the effects of Elp3 on the regulation of fungal growth and conidiation, RNA-seq analysis revealed that the Δ*elp3* mutant strain displayed reduced expression of GAG biosynthesis cluster genes compared to the wild-type strain (Fig 3). GAG is an exopolysaccharide that is considered to be the key virulence factor that mediates fungal adhesion, biofilm formation and host immune recognition mechanisms [39]. Similarly, deletion of *elp3* resulted in reduced GAG production and adherence to polystyrene plates. The GAG biosynthesis cluster is composed of five genes located on chromosome 3 in *A*. *fumigatus* [26], and these genes include *uge3*, which encodes a glucose 4-epimerase; *agd3*, which encodes a secreted polysaccharide deacetylase; *gtb3*, which encodes a putative transmembrane glycosyltransferase; and *ega3* and *sph3*, which encode two glycoside hydrolases [40]. It has been demonstrated that the transcription factor SomA, its interacting protein PtaB, and the regulators of fungal development MedA and StuA positively regulate the expression of GAG cluster genes [39,41–43]. In the Δ*elp3* mutant, we did not observe any significant changes in the expression of these GAG regulons at either the mRNA or protein levels (S11 Fig). Therefore, Elp3 may affect GAG cluster gene expression in a SomA-independent manner. In addition, we noticed that the correlation between proteomic and transcriptomic data is rather low (R = 0.1423) (S12 Fig), suggesting that the transcriptional changes observed in the RNA-seq results are most likely secondary effects. Importantly, we have also demonstrated that *A*. *fumigatus* Elp3 is critical for virulence in both wax moth and murine models (Fig 4G–4I). These results were consistent with the observation in the phytopathogen *Pyricularia oryzae* [34], where the decreased pathogenicity may be attributed to hyperactivation of autophagy.

The well-defined structures of the Elongator complex and its subunits from yeast and bacterial species facilitate our understanding of the underlying regulatory mechanism [11,15,44,45]. Since Elp3 proteins from different species have high protein sequence homology, we built a high-confidence structure of *A*. *fumigatus* Elp3 using *S*. *cerevisiae* Elp3 as a template. Comparison of the structures revealed that C129 and C132 in the rSAM domain and Y551 and Y552 in the KAT domain, which coordinate to form the Fe-S cluster and desulfo-CoA, respectively, are critical for Elp3 function (Fig 5); these results indicate that both the rSAM and KAT domains play indispensable roles. However, we have to point out that the current conclusion is only based on the observation that the expression of these mutations was unaffected at the mRNA level (S5 Fig), it is not excluded that the protein levels could be changed. Elp3 was originally considered to be involved in the facilitation of elongation and to play an important role in transcription by exerting its acetyltransferase effects on lysine 14 of histone H3, as it was initially copurified from the elongating form of RNAPII and contains a KAT domain [17]. Although our lysine acetylation assay showed that Elp3 is capable of acetylating H3K14 *in vitro*, the acetylation levels of H3K14 remained unchanged in the Δ*elp3* mutant strain (Fig 6A–6C). These results suggested that other lysine acetyltransferases can compensate for the loss of Elp3 in *A*. *fumigatus*.

Indeed, the regulatory mechanism of the Elongator complex has been under extensive debate [32]. Increasing evidence has recently demonstrated that the primary function of Elp3 is to modify tRNAs at the U_34_ wobble base [9], as all the defective phenotypes of the *elp3* null mutant strain result from loss of U_34_ modification. The *elp3* null mutant in yeast displays severely decreased levels of mcm^5^s^2^U and increased levels of two types of tRNA (tRNA^Lys^_UUU_ and tRNA^Gln^_UUG_), which is assumed to counteract translational deficiency during the codon recognition process and to suppress growth abnormalities [24,46]. In *A*. *fumigatus*, the predominant cytoplasmic localization of Elp3 further suggested that Elp3 may participate in the tRNA modification that takes place in the cytoplasm rather than in the histone acetylation that takes place in the nucleus (Fig 1C and 1D). Here, we present evidence demonstrating that Elp3 is required for the mcm^5^s^2^ modification of wobble uridines of tRNAs in *A*. *fumigatus* (Fig 6D–6F), supporting its conserved function in tRNA modification in eukaryotes. This conserved function was further confirmed by the results showing that the overexpression of tRNAs could restore the defects in hyphal growth, GAG production and adhesion observed in the Δ*elp3* mutant strain (Figs 6G, 6H and S7). Therefore, our results provide clear evidence that the phenotypes in the Δ*elp3* mutant strain arise as a consequence of defects in U_34_ modification. Interestingly, we observed that tRNA overexpression only partially suppressed these phenotypes, and the degree of suppression varied among different fungal species. tRNA^Gln^_UUG_ was the most effective in suppressing these phenotypes in *A*. *fumigatus*, while tRNA^Lys^_UUU_ was more efficient in yeast, suggesting that the dependence of Elp3-mediated U_34_ tRNA modification may be different between yeast and *A*. *fumigatus*.

Mechanistically, previous studies showed that the phenotypes associated with defective U_34_ modification were primarily attributed either to disrupted protein homeostasis triggered by proteotoxic stress via the unfolded protein response [28,47] or to reduced expression of particular proteins whose mRNA is enriched in UUU, UUC and UUG [29,30,48]. However, we did not observe a robust unfolded protein response in the Δ*elp3* mutant strain based on our transcriptome and proteome data (S9 Fig). In addition, codon usage analysis revealed no significant AA-ending codon bias in the genes encoding the downregulated proteins (S8 Fig). These results suggested that Elp3 in *A*. *fumigatus* might regulate fungal growth, GAG production and adhesion through other mechanisms. Recently, the findings in yeast have revealed associations between U_34_ modification and the cellular metabolic state [49]. U_34_ tRNA modification is considered to be a metabolic cue for the availability of sulfur amino acids. Its deficiency resulted in rewired carbon and nitrogen metabolism and triggered the amino acid starvation pathway mediated by the transcription factor Gcn4 [49,50]. Ultimately, the altered metabolic state affects the growth of mutant strains with defective U_34_ tRNA modification. Consistent with this hypothesis, we observed the overexpression of CpcA, a homolog of *S*. *cerevisiae* Gcn4, in the Δ*elp3* mutant strain (Fig 7C and 7D). More strikingly, deletion of the *cpcA* gene significantly rescued the defective hyphal growth, reduced GAG production and attenuated virulence of the *elp3* null mutant strain (Fig 7E–7J), as well as oxidative stress resistance (S10 Fig). These results strongly indicated that the metabolic imbalance caused by tRNA modification deficiency was a major contributor to the defective phenotypes of the Δ*elp3* mutant strain. In addition, we noticed that deletion of *cpcA* alone did not significantly affect the virulence of *A*. *fumigatus* in our models (Fig 7E–7J), which is in contrast to previous reports [51]. These contrasting results may be attributed to differences in the genetic backgrounds of the strains or immunosuppressive mouse model used. However, the reason for this discrepancy remains to be further investigated.

It is generally believed that certain mutants with enhanced susceptibility to oxidative stress tend to be less virulent *in vivo* since host-generated ROS kills fungal pathogens directly by causing oxidative damage. However, our results showed that the Δ*elp3* mutant was less virulent in both wax moth and murine models but had increased resistance to oxidative stress (Figs 4G, 4H and S10), which showed an inverse correlation. Actually, the susceptibility to oxidative stress *in vitro* is not always correlated with reduced virulence in animal models. For example, deletion of the *A*. *fumigatus* redox-responsive transcription factor Yap1 results in drastically increased sensitivity to H_2_O_2_ and menadione but had no impact on virulence in a murine model [52]. A similar observation was also reported in the superoxide dismutase family in *A*. *fumigatus* [53]. Moreover, our results showed that the increase of GAG production by overexpression of *uge3* or *agd3* could also partially restore virulence of the Δ*elp3* mutant in both wax moth and murine models (Fig 7I and 7J), suggesting that the attenuated virulence of the *A*. *fumigatus* Δ*elp3* mutant strain arise as a consequence of amino acid homeostasis imbalance related-growth defects and reduced GAG production and adhesion, but not an altered response to oxidative stress. In addition, we cannot exclude the possibility that Elp3 performs its functions by acetylating nonhistone substrates, as neither tRNA overexpression nor CpcA inhibition fully suppressed the phenotypes of the Δ*elp3* mutant strain.

In conclusion, the present work reveals a critical role of the tRNA-modifying enzyme Elp3 in the regulation of hyphal growth, GAG production, adhesion and virulence in *A*. *fumigatus*. More broadly, these findings provide important insight into the mechanism underlying Elp3-mediated U_34_ tRNA modifications in fungal pathogens.

## Materials and methods

### Ethics statement

Animal infection experiments were performed following the protocols approved by the Animal Care and Use Committee of Nanjing Normal University, China (permit no. LACUC-20200703).

### Strains and culture conditions

The *A*. *fumigatus* strains used in this study are listed in S1 Table. All the strains were grown on minimal medium (MM), which contained 10 g/liter glucose, 1 ml/liter trace elements, 50 ml/liter 20 × salt solution and 2% agar, or yeast extract and glucose (YG), which contained 20 g/liter glucose, 1 ml/liter trace elements, and 5 g/liter yeast extract. The spores were harvested from cultures on solid YG by using 0.02% (vol/vol) Tween 20 in a saline solution. *A*. *fumigatus* Δ*KU80* was used as the wild-type control strain [54].

### Construction of the *A*. *fumigatus elp3* mutants

The primer sequences utilized in this work are listed in S2 Table. The full open reading frame (ORF) of *elp3* was replaced by the nutrition marker *pyr4* to create an *elp3*-null mutant strain. Using the primers Elp3 P2/P5, the upstream and downstream homologous portions of the target gene and the resistance gene marker were fused to create the *elp3* replacement cassette. The Elp3 P1/P3 and Elp3 P4/P6 primers were used to amplify the flanking fragments from *A*. *fumigatus* wild-type genomic DNA by PCR, and the 2.1-kb *pyr4* gene was amplified from the plasmid pAL5 using the primers pyr4-F and pyr4-R. The other Elongator complex subunit-knockout mutants were constructed following methods similar to those used to construct the *elp3* null strain. *A*. *fumigatus* A1160 was used as the recipient strain for transformation.

Reintroducing a full-length *elp3* gene into the *elp3*-null mutant strain, including its native promoter, 5’ UTR, ORF, and 3’ UTR, allowed for genetic complementation. With the primers Elp3-F/Elp3-R, the *elp3* gene fragment was amplified from wild-type *A*. *fumigatus* genomic DNA and then fused to a resistance marker, hygromycin B phosphotransferase (*hph*), with the primers Elp3-F and hph-R. With the primers hph-F and hph-R, the *hph* gene was amplified from the plasmid pAN7-1. The resultant DNA fragment was fused with *hph* and subsequently transferred to *the elp3*-null mutant strain.

To construct the recombinant Elp3-GFP strain, the upstream and downstream regions were also amplified using the primers Elp3-GFP P1/P3 and Elp3-GFP P4/6, respectively. The termination codon of the *elp3* ORF was located outside the upstream sequence. Using the primers GFP-pyrG F/R, a fragment containing GFP and the selectable gene *AfpyrG* was amplified from the plasmid pFNO3. The three PCR products were then fused with the Elp3-GFP P2/P5 primers and transformed into the A1160 recipient strain. The Elp3-FLAG recombinant strain was constructed following methods similar to those used to construct the Elp3-GFP recombinant strain.

The *elp3* null mutant was utilized as the recipient strain, and then, a distinct point mutation in *elp3* was introduced to create point mutation strains. The template was the plasmid bearing the reintroduced *elp3* fragment from the genetic complementation experiment. The desired mutation was created and synthesized into primers. Linearized DNA fragments containing point mutations were generated by PCR using the corresponding primer pairs, and the template plasmid was then digested with DpnI for 2 hours at 37°C. The DNA products were subcloned into pEASY-Blunt zero (TransGen Biotech) and subsequently transformed into Trans1-T1 *E*. *coli* (TransGen Biotech). Sequencing revealed the presence of the desired mutation in all the plasmids.

The transformation of *A*. *fumigatus* was carried out as previously described [55]. The selection marker hygromycin B was used to select transformants at a concentration of 200 μg/ml.

### Quantification of conidia

To quantify production of conidia, spores (2 × 10^4^) of the indicated strains were inoculated onto solid minimum media (3.5-cm-diameter Petri plate). After 48 h of incubation at 37°C, conidia were harvested with 2 ml distilled water containing 0.02% Tween 20 and quantified using a hemocytometer under a bright-field microscope.

### Fluorescence microscopy

A total of 1 × 10^6^ spores of the Elp3-GFP strain were cultured on glass coverslips in 1 ml liquid MM for 12 hours to visualize the localization of the Elp3-GFP fusion protein. The medium was removed, and the samples were washed three times in phosphate-buffered saline (PBS). The samples were then fixed for 30 minutes at room temperature with 4% (vol/vol) formaldehyde. Then, the samples were washed three times with PBS and incubated for 60 minutes at room temperature in the dark with Hoechst 33258 solution (Sangon Biotech, E607301) at a final concentration of 1 μg/ml. GAG was stained with the specific lectin SBA and visualized by fluorescence microscopy as previously described [56]. The mean fluorescence intensity (MFI) of SBA-FITC from three random pictures taken from three independent experiments was quantified by Image J software. A Zeiss Axio imager A1 microscope was used to collect all of the images (Carl Zeiss, Jena, Germany).

### Adherence assays

The adherence assay was performed as previously described with minor modifications [57]. The *A*. *fumigatus* strains were cultivated in 96-well polystyrene plates at 37°C for 18 hours in RPMI 1640 medium. Then, the strains were washed twice with 100 μl of H_2_O to eliminate nonadherent fungi. Adherent biofilms were visualized by staining with 100 μl of 0.1% (w/v) crystal violet. After removing the crystal violet solution, each well was washed twice with 300 μl of H_2_O. Adherent biofilms were then destained by adding 125 μl ethanol, and the ethanol was transferred to a clean 96-well microtiter plate to measure the OD_600_ using a microtiter plate reader.

### Proteomics

TMT-based proteomics analysis was performed by Luming Biotech (Shanghai, China). Total proteins were extracted using a Tris-buffered phenol method. A total of 50 μg protein from each sample was digested with trypsin for TMT labeling and proteomic analysis. The TMT-labeled peptides were fractionated by 1100 HPLC System (Agilent) using an Agilent Zorbax Extend RP column (5 μm, 150 mm × 2.1 mm). The LC-MS/MS analysis was performed using a Triple TOF 5600 mass spectrometer equipped with a Nanospray III source (SCIEX, USA). The MS raw data were processed with MaxQuant and searched against *Aspergillus fumigatus* A1163 database in FungiDB (https://fungidb.org/fungidb/). Proteomic data are available on ProteomeXchange under accession number PXD033087.

### Quantitative real-time PCR and RNA-sequencing

Total RNA was obtained for qRT–PCR using a spin column fungal total RNA purification kit (Sango Biotech) following the manufacturer’s directions. After digesting the genomic DNA, 500 ng RNA was used to synthesize cDNA using the HiScript TM II QRT Super Mix for qPCR kit (Vazyme, R233). qRT–PCR was carried out using AceQ Universal SYBR qPCR Master Mix (Vazyme, Q511) on an ABI Onestep rapid thermocycler (Applied Biosystems). The fold expression was computed using the 2^-ΔΔCt^ method, and the expression was normalized to tubulin. RNA sequencing of the wild-type and *elp3*-null mutant was performed on the Illumina platform (Personalbio, Shanghai, China). The RNA-seq data were deposited in the NCBI SRA (sequence read archive) database under accession number PRJNA823553.

### tRNA modification analysis

LC–MS-based tRNA modification analysis was performed by Aksomics (Shanghai, China). Total RNA samples were separated by agarose gel electrophoresis and quantified using a Nanodrop. tRNA was isolated from total RNA by the urea-PAGE method, followed by hydrolysis to single nucleosides and dephosphorylation. Purified tRNA was digested to single dephosphorylated nucleosides by an enzyme mix. LC–MS analysis was performed on an Agilent 6460 QQQ mass spectrometer with an Agilent 1260 HPLC system using multi-reaction monitoring (MRM) detection mode. LS-MS data were acquired using Agilent Qualitative Analysis software. The MRM peaks of each modified nucleoside were extracted and normalized to the quantity of tRNA purified from each sample.

### Western blotting analysis

The indicated strains were inoculated into liquid MM and cultivated for 24 hours at 37°C and 200 rpm. With a mortar and pestle, the mycelia were gathered and homogenized in liquid nitrogen. The protein extracts were obtained as previously described [58]. The nuclear and cytoplasmic fractions were extracted by using a Nuclear and Cytoplasmic Protein Extraction Kit (Beyotime, P0027). SDS–PAGE gels were used to separate the total protein, which was then transferred to polyvinylidene difluoride (PVDF) membranes (Millipore). Anti-mouse FLAG (1:5000, Sigma–Aldrich, F3165), anti-rabbit H3 (1:5000, Sigma–Aldrich, H9289), anti-mouse GFP (1:2000, Roche, 11814460001) and anti-rabbit H3K14ac (1:5000, Abclonal, A7254) were used to probe the blots. The secondary antibodies were peroxidase-labeled goat anti-mouse (1:5000, ABclonal, AS003) and goat anti-rabbit (1:5000, ABclonal, AS014). The Enhanced ECL luminescence detection kit (Vazyme, E411) was used to visualize blots, and images were captured with a Tanon 4200 chemiluminescent imaging system. ImageJ software was used to calculate the band intensities.

### Elp3 FLAG pull down

Elp3-FLAG strain protein extracts were prepared by extracting homogenized mycelia with lysis buffer (10 mM Tris-HCl pH 7.5, 150 mM NaCl, 0.5 mM EDTA, 0.01% Triton X-100, 1 mM DTT, 1 mM PMSF, and protease inhibitor mixture). The total protein contents of the crude extracts were determined using the Bradford method, and 5 mg of protein was gently mixed with the ANTI-FLAG M2 Affinity gel (Sigma–Aldrich, A2220) before purification according to the manufacturer’s recommendations. BGI Genomics performed the liquid chromatography–tandem mass spectrometry.

### *In vitro* lysine acetylation assays

Elp3 protein was purified from the Elp3-FLAG strain using an anti-FLAG-M2 agarose column (Sigma–Aldrich) as described above. The *in vitro* KAT assay was carried out as previously described [59]. In a total volume of 25 μl, highly purified HeLa H3/H4 core histones were incubated with 2.5 μg acetyl-CoA (Sigma–Aldrich) and 10 μg purified Elp3 in HAT reaction buffer (50 mM Tris-HCl pH 8.0, 100 mM NaCl, 5 mM MgCl_2_, 1 mM dithiothreitol). After 1 hour of incubation at 30°C, the mixture was treated with 2 × SDS loading dye and heated at 95°C for 10 minutes. The acetylation levels in these samples were determined using western blotting.

### Virulence assay

In the *G*. *mellonella* model, ten microliters of indicated *A*. *fumigatus* strains were injected into *G*. *mellonella* larvae (approximately 0.3 g) via the left prolegs. As a control group, larvae were injected with PBS. All the larvae were incubated for up to 7 days at 37°C in the dark, and their survival was evaluated every 24 hours. The procedures were conducted three times with groups of twenty larvae per sample.

Murine infection assays were performed as previously described with slight modifications [60]. Eight-week-old female C57BL/6 mice were immunosuppressed with 150 mg/kg cyclophosphamide on days -4 and -1. On Day 0, the mice were sedated with isoflurane and subsequently injected with 5 × 10^6^ *A*. *fumigatus* conidia in 30 μl of PBS by intratracheal instillation, and PBS alone was used as a control. After infection, 75 mg/kg cyclophosphamide was administered every three days to maintain immunosuppression. Animal body weights were measured daily, and the mice were sacrificed when their weight had dropped by 20% compared with the baseline weight. For the survival assay, the mice were monitored twice daily for 10 days to assess morbidity and mortality, ten mice were infected for each group. The statistical significance of survival curves was calculated using the log-rank test. For histological examination, the mice were killed 72 h after infection, and lung tissues were harvested for Grocott’s methenamine silver (GMS) or hematoxylin and eosin (HE) staining following standard protocols.

## Supporting information

S1 Fig Sequence alignment and phylogenetic analysis of Elp3 orthologs in other species. (A) Protein sequence alignment was performed using the Clustal Omega based on Clustal W multiple sequence alignment method. rSAM and KAT domains are colored in blue and pink, respectively. The figure was drawn with ESPript3. (B) Phylogenetic analysis was performed on the Phylogeny.fr platform (http://www.phylogeny.fr) using the maximum likelihood method. Species names are shown in the figure followed by the GenBank accession number.
(TIF)

S2 Fig Construction of the Elongator complex subunit deletion mutants. (A) Diagram illustrating the targeted gene homologous replacement for the Elongator complex subunit gene. (B) Diagnostic PCR confirmed the homologous integration at the original locus in the deletion strains.
(TIF)

S3 Fig Colony morphologies of Elp3-GFP and Elp3-FLAG strains grown on minimal media. (A) Growth phenotypes of Elp3-GFP and Elp3-FLAG strains on solid minimal medium at 37°C for 48 h. (B) Quantitative examination of the diameters of the colonies formed by the indicated strains. The data are presented as the mean ± SEM (standard error of the mean) of three independent experiments. Statistical analysis was performed using one-tailed, unpaired *t* tests. **p < 0.01; ns, not significant.
(TIF)

S4 Fig *elp3* point mutation strains exhibit reduced GAG production and adhesion. (A) Quantification of adhesion ability in the indicated strains by crystal violet staining. The data are presented as the mean ± SEM (standard error of the mean) of three independent experiments. Statistical analysis was performed using one-tailed, unpaired *t* tests. **p < 0.01. (B) GAG production of the indicated strains was visualized by soybean agglutinin (SBA) lectin staining. Scale bar = 10 μm. The mean fluorescence intensity (MFI) of SBA-FITC in the indicated strains was calculated and normalized to the wild-type (100%).
(TIF)

S5 Fig mRNA expression of point mutations in the Δ*elp3* strains. Expression analysis of each point mutation in liquid MM by qRT-PCR. The mRNA levels were normalized to an mRNA level of the reference gene *tubA*. The data are presented as the mean ± SEM (standard error of the mean) of three independent experiments. Statistical analysis was performed using one-way ANOVA with multiple comparisons tests. ns, not significant.
(TIF)

S6 Fig The expression of GAG cluster genes and GAG production was not affected in the H3K14R mutant strain. (A) Colony morphology of the wild-type, Δ*elp3* and H3K14R mutants grown on solid minimum medium MM and complete YG medium at 37°C for 48 h. (B) Quantitative real-time RT-PCR analysis of GAG cluster genes in the wild-type and H3K14R strains. The mRNA levels were normalized to the reference gene *tubA*. The data are presented as the mean ± SEM (standard error of the mean) of three independent experiments. Statistical analysis was performed using one-tailed, unpaired *t* tests. ns, not significant. (C) GAG production of the wild-type, Δ*elp3* and H3K14R strains was visualized by soybean agglutinin (SBA) lectin staining. Scale bar = 10 μm. The mean fluorescence intensity (MFI) of SBA-FITC in the indicated strains was calculated and normalized to the wild-type (100%).
(TIF)

S7 Fig Colony diameter quantification and GAG cluster gene expression in the Δ*elp3* mutant overexpressing corresponding tRNAs. (A) Quantitative analysis of colony diameter of the indicated strains grown on MM and YG at 37°C for 48 h. The data are presented as the mean ± SEM (standard error of the mean) of three independent experiments. Statistical analysis was performed using one-tailed, unpaired *t* tests. **p < 0.01; ns, not significant. (B) Quantitative real-time RT-PCR analysis of GAG cluster genes in the wild-type, Δ*elp3* and tQ^UUG^ strains. The mRNA levels were normalized to the reference gene *tubA*. The data are presented as the mean ± SEM (standard error of the mean) of three independent experiments. Statistical analysis was performed using one-tailed, unpaired *t* tests. **p < 0.01; ns, not significant.
(TIF)

S8 Fig Heatmap of normalized codon frequencies of AAA, CAA, and GAA in the downregulated proteins of the Δ*elp3* mutant. (TIF)

S9 Fig Heatmap of protein folding related gene expressions in the Δ*elp3* mutant compared to the wild-type. (TIF)

S10 Fig The Δ*elp3* mutant exhibits increased resistance to oxidative stress. (A) Colony morphology of the wild-type, Δ*elp3*, Δ*elp3*Δ*cpcA*, Δ*cpcA* and complementation strains grown on solid MM in the presence of 4 mM H_2_O_2_ and 10 μM menadione at 37°C for 48 h. (B) Relative hyphal growth inhibition of the indicated strains at 37°C for 48 h. The data are presented as the mean ± SEM (standard error of the mean) of three independent experiments. Statistical analysis was performed using one-tailed, unpaired t tests. **p < 0.01; ns, not significant.
(TIF)

S11 Fig The protein expression of SomA, PtaB and MedA is not affected in the Δ*elp3* mutant. (A) Schematic diagram of GAG biosynthesis pathway. (B) Quantitative real-time RT-PCR analysis of *somA*, *ptaB* and *medA* in the wild-type and Δ*elp3* strains. The mRNA levels were normalized to the reference gene *tubA*. The data are presented as the mean ± SEM (standard error of the mean) of three independent experiments. Statistical analysis was performed using one-tailed, unpaired *t* tests. ns, not significant. (C) Western blots show the protein expression of SomA, PtaB and MedA in the wild-type and Δ*elp3* strains. β-actin served as the loading control.
(TIF)

S12 Fig Correlation between log_2_ fold changes in mRNA and protein levels in the wild-type and Δ*elp3* strains. Common significant regulation at mRNA and protein levels is indicated in red (upregulation) and blue (downregulation), respectively. R, Pearson correlation coefficient.
(TIF)

S1 Table Strains used in this study. (DOCX)

S2 Table Primers used in this study. (DOCX)

S3 Table All putative Elp3-interacting proteins identified by the FLAG pull-down assay. (XLS)

S4 Table All differently expressed genes in the wild-type and Δ*elp3* strains. (XLSX)

S5 Table All differently expressed proteins in the wild-type and Δ*elp3* strains. (XLSX)
