# Supplementary figures and images for: Aspergillus fumigatus Elongator complex subunit 3 affects hyphal growth, adhesion and virulence through wobble uridine tRNA modification
Source: PLoS Pathog. 2022 Nov 14;18(11):e1010976. doi: 10.1371/journal.ppat.1010976 (PMC9704764; doi:10.1371/journal.ppat.1010976)

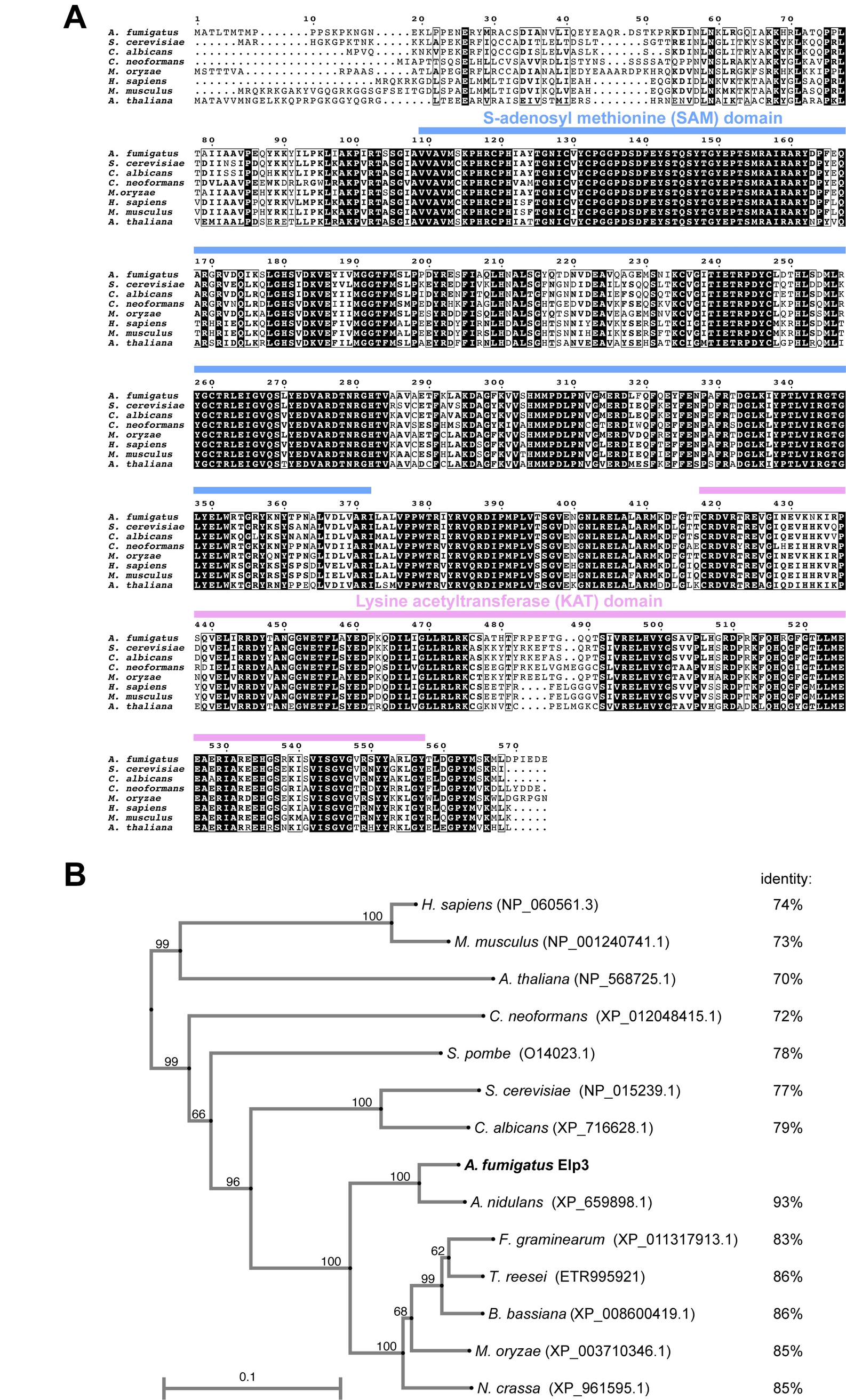

Supplement: S1 Fig — (A) Protein sequence alignment was performed using the Clustal Omega based on Clustal W multiple sequence alignment method. rSAM and KAT domains are colored in blue and pink, respectively. The figure was drawn with ESPript3. (B) Phylogenetic analysis was performed on the Phylogeny.fr platform (http://www.phylogeny.fr) using the maximum likelihood method. Species names are shown in the figure followed by the GenBank accession number. (TIF) [file ppat.1010976.s001.tif]

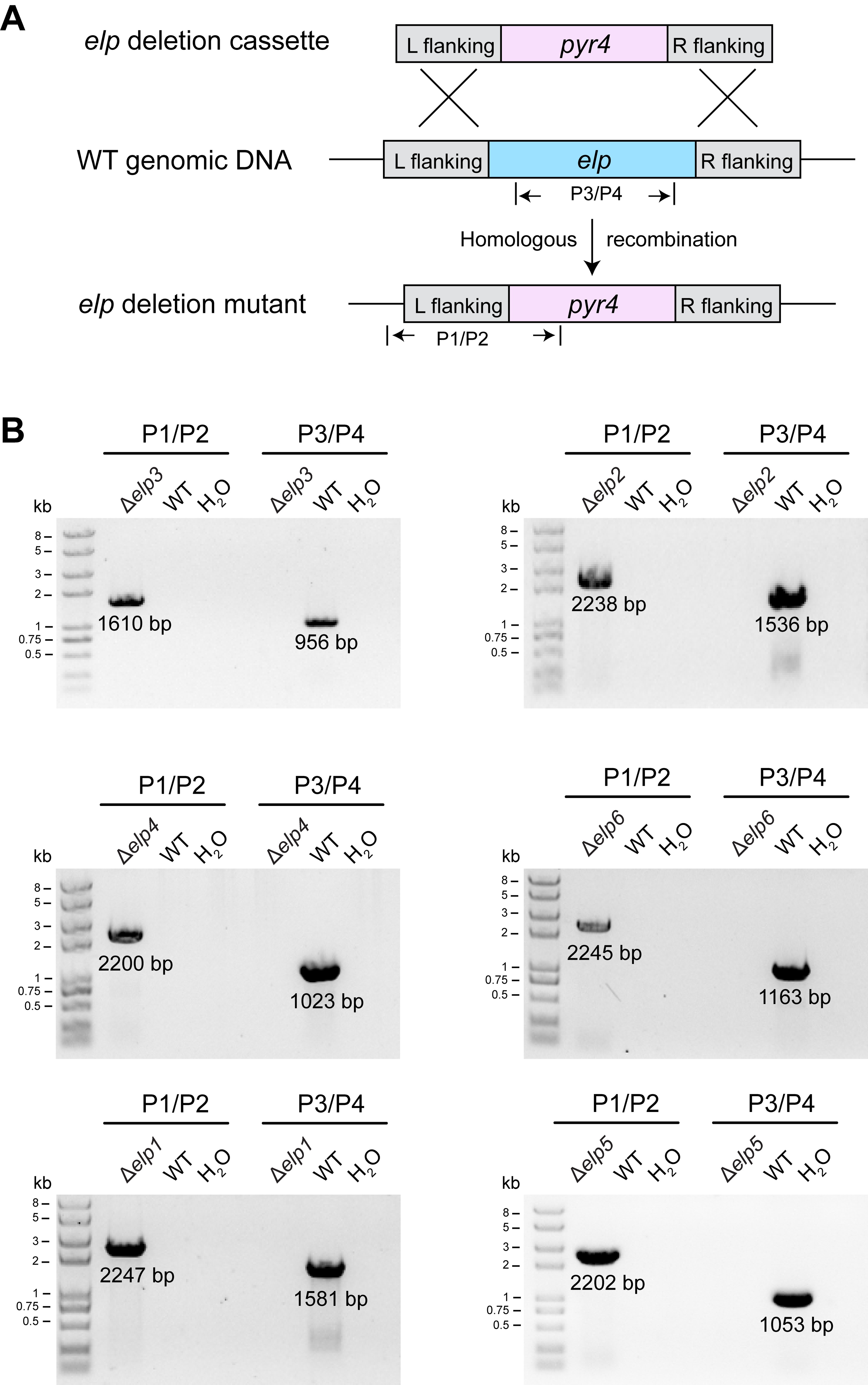

Supplement: S2 Fig — (A) Diagram illustrating the targeted gene homologous replacement for the Elongator complex subunit gene. (B) Diagnostic PCR confirmed the homologous integration at the original locus in the deletion strains. (TIF) [file ppat.1010976.s002.tif]

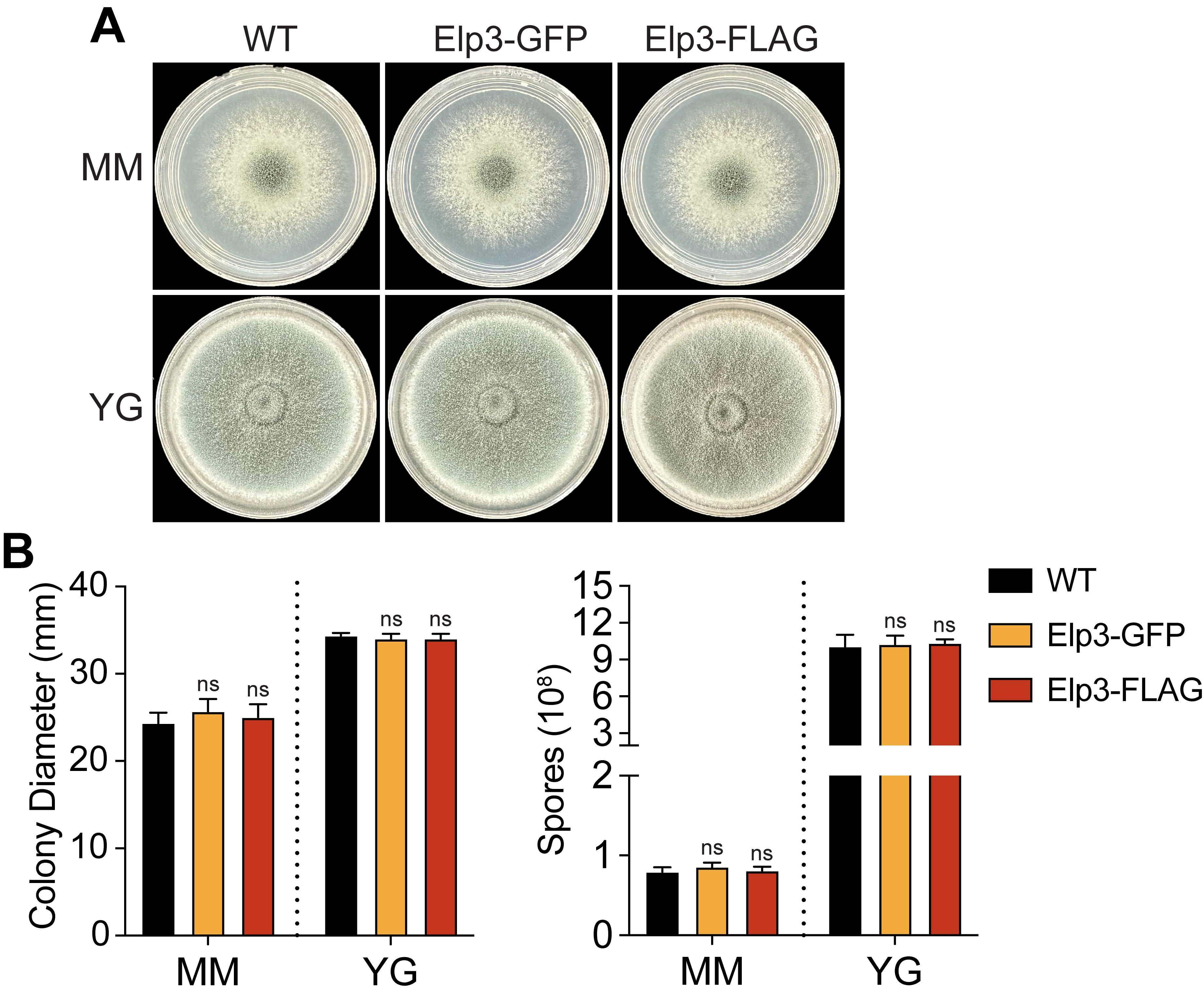

Supplement: S3 Fig — (A) Growth phenotypes of Elp3-GFP and Elp3-FLAG strains on solid minimal medium at 37°C for 48 h. (B) Quantitative examination of the diameters of the colonies formed by the indicated strains. The data are presented as the mean ± SEM (standard error of the mean) of three independent experiments. Statistical analysis was performed using one-tailed, unpaired t tests. **p < 0.01; ns, not significant. (TIF) [file ppat.1010976.s003.tif]

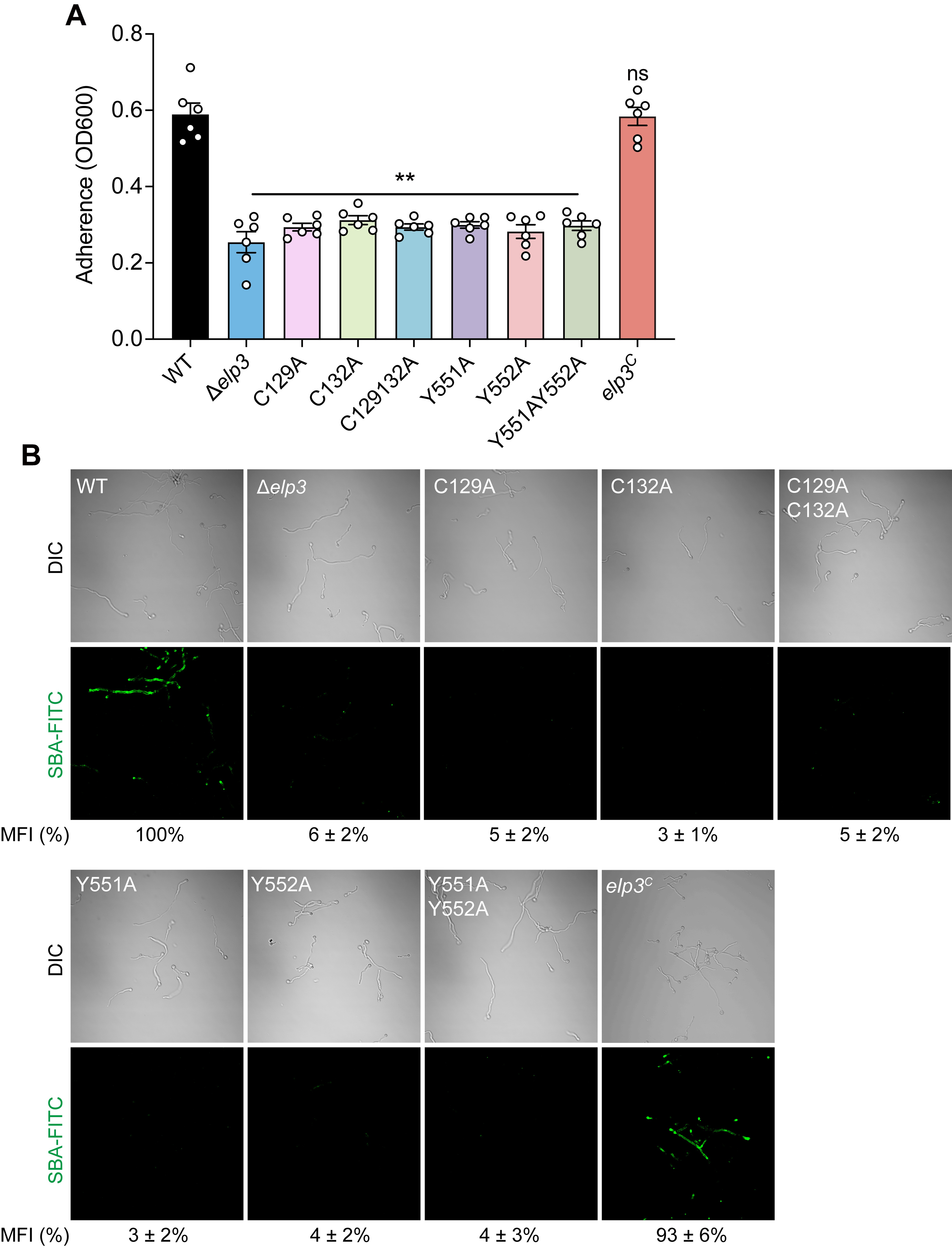

Supplement: S4 Fig — (A) Quantification of adhesion ability in the indicated strains by crystal violet staining. The data are presented as the mean ± SEM (standard error of the mean) of three independent experiments. Statistical analysis was performed using one-tailed, unpaired t tests. **p < 0.01. (B) GAG production of the indicated strains was visualized by soybean agglutinin (SBA) lectin staining. Scale bar = 10 μm. The mean fluorescence intensity (MFI) of SBA-FITC in the indicated strains was calculated and normalized to the wild-type (100%). (TIF) [file ppat.1010976.s004.tif]

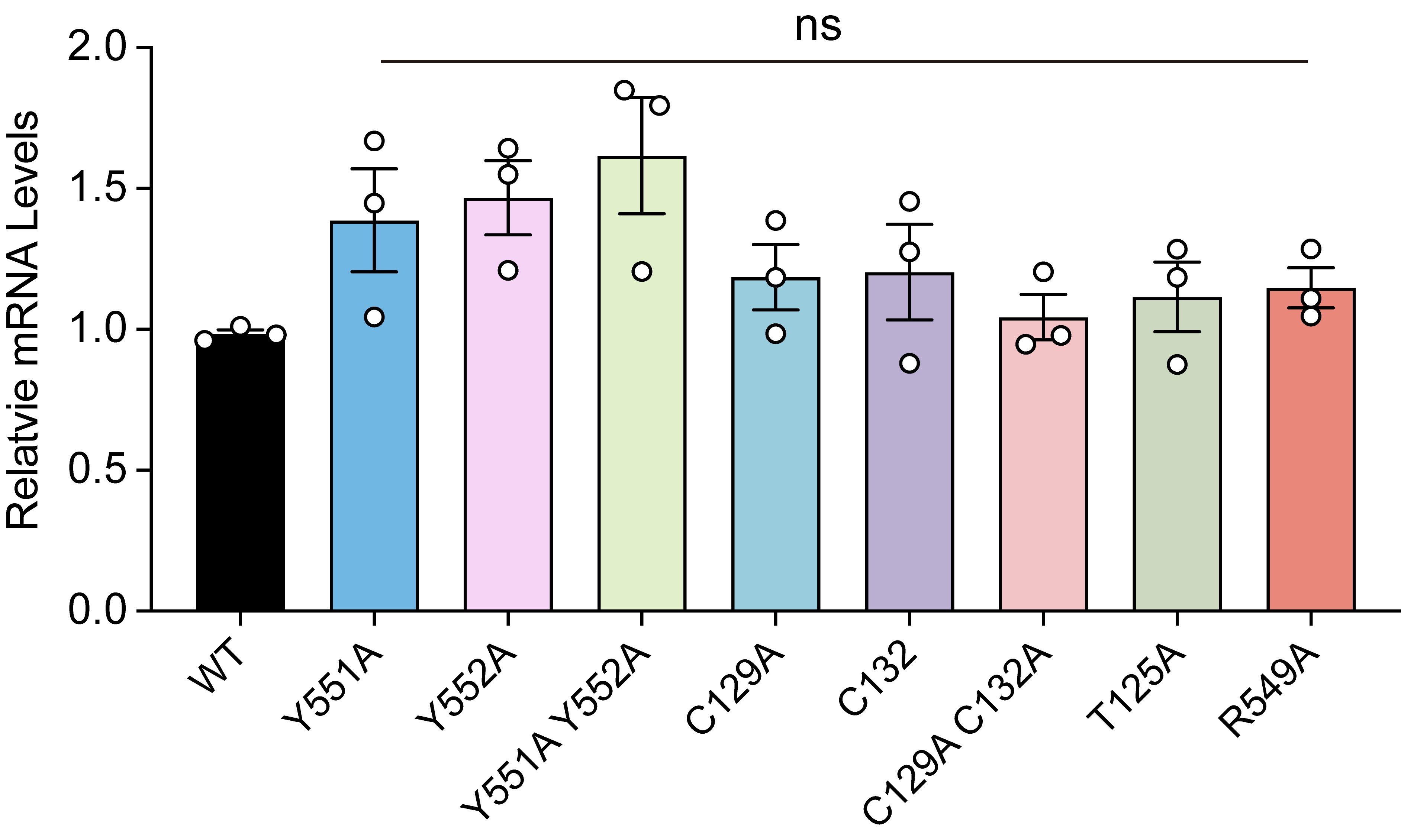

Supplement: S5 Fig — Expression analysis of each point mutation in liquid MM by qRT-PCR. The mRNA levels were normalized to an mRNA level of the reference gene tubA. The data are presented as the mean ± SEM (standard error of the mean) of three independent experiments. Statistical analysis was performed using one-way ANOVA with multiple comparisons tests. ns, not significant. (TIF) [file ppat.1010976.s005.tif]

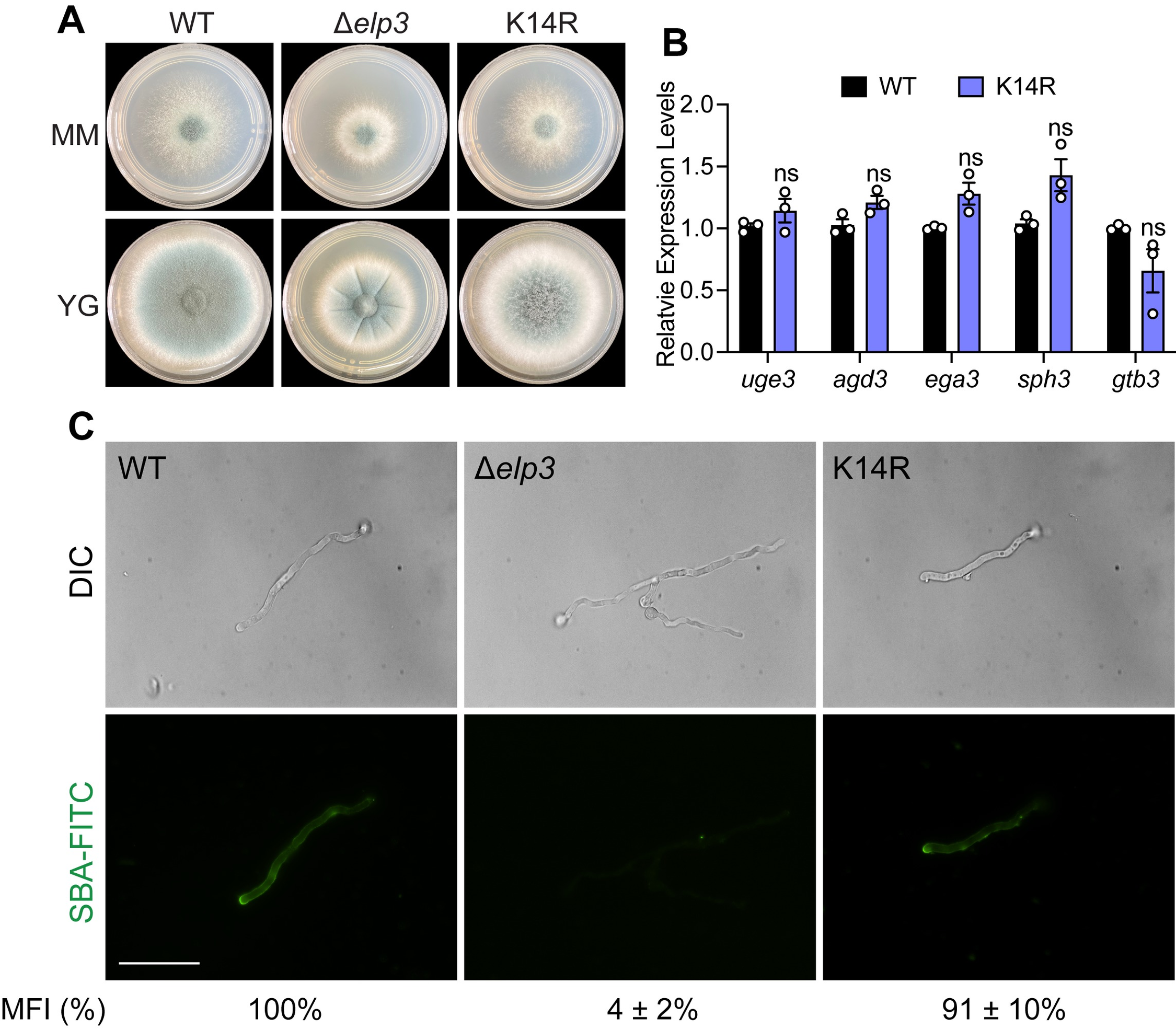

Supplement: S6 Fig — (A) Colony morphology of the wild-type, Δelp3 and H3K14R mutants grown on solid minimum medium MM and complete YG medium at 37°C for 48 h. (B) Quantitative real-time RT-PCR analysis of GAG cluster genes in the wild-type and H3K14R strains. The mRNA levels were normalized to the reference gene tubA. The data are presented as the mean ± SEM (standard error of the mean) of three independent experiments. Statistical analysis was performed using one-tailed, unpaired t tests. ns, not significant. (C) GAG production of the wild-type, Δelp3 and H3K14R strains was visualized by soybean agglutinin (SBA) lectin staining. Scale bar = 10 μm. The mean fluorescence intensity (MFI) of SBA-FITC in the indicated strains was calculated and normalized to the wild-type (100%). (TIF) [file ppat.1010976.s006.tif]

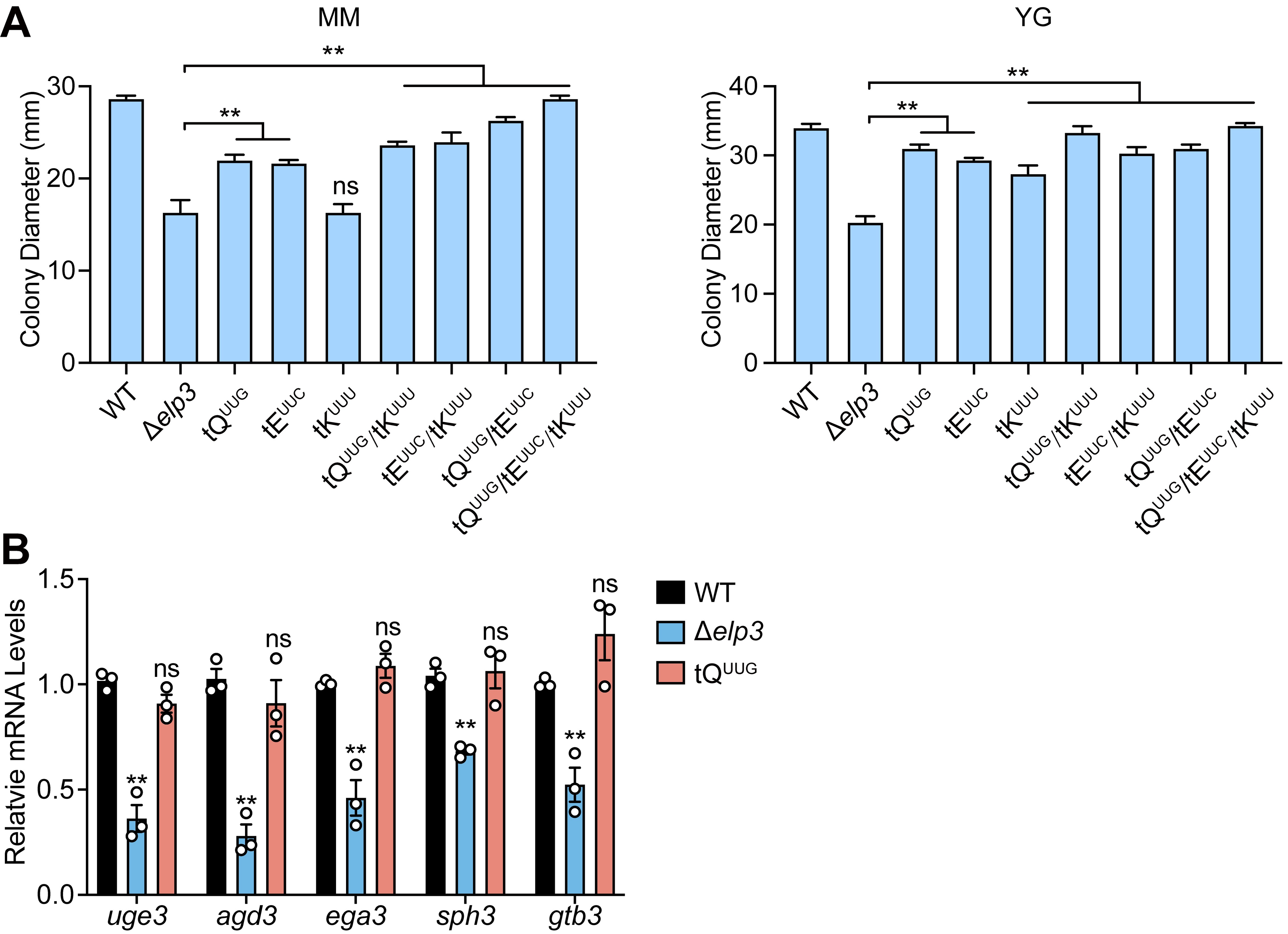

Supplement: S7 Fig — (A) Quantitative analysis of colony diameter of the indicated strains grown on MM and YG at 37°C for 48 h. The data are presented as the mean ± SEM (standard error of the mean) of three independent experiments. Statistical analysis was performed using one-tailed, unpaired t tests. **p < 0.01; ns, not significant. (B) Quantitative real-time RT-PCR analysis of GAG cluster genes in the wild-type, Δelp3 and tQUUG strains. The mRNA levels were normalized to the reference gene tubA. The data are presented as the mean ± SEM (standard error of the mean) of three independent experiments. Statistical analysis was performed using one-tailed, unpaired t tests. **p < 0.01; ns, not significant. (TIF) [file ppat.1010976.s007.tif]

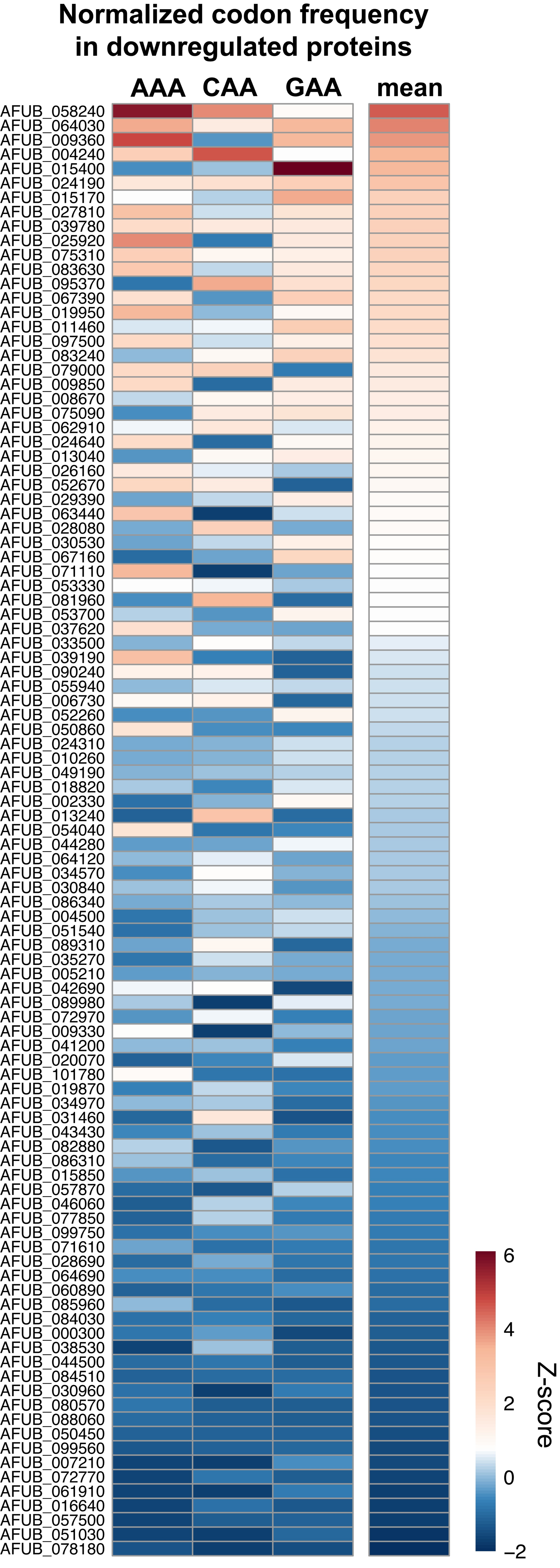

Supplement: S8 Fig — (TIF) [file ppat.1010976.s008.tif]

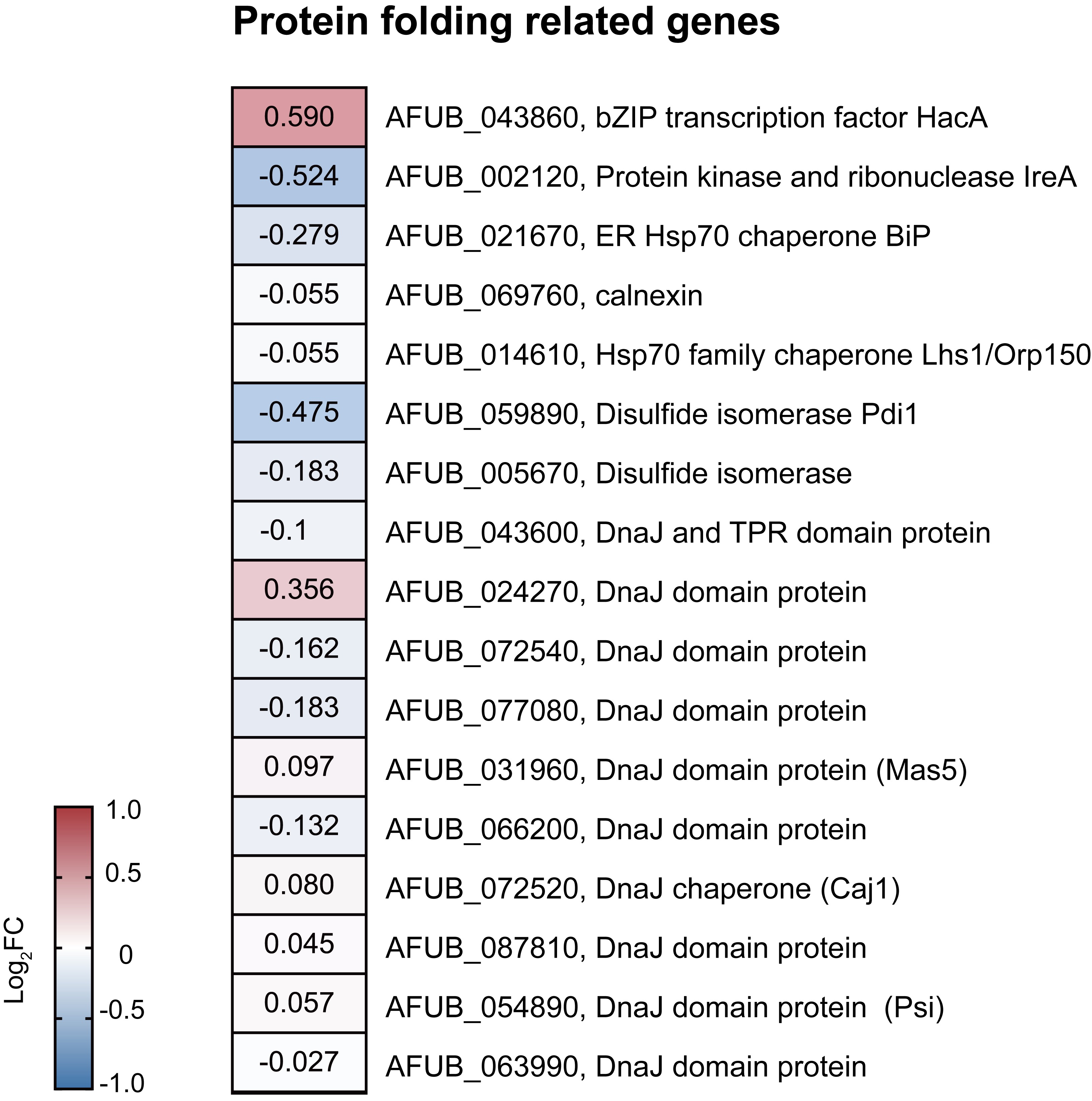

Supplement: S9 Fig — (TIF) [file ppat.1010976.s009.tif]

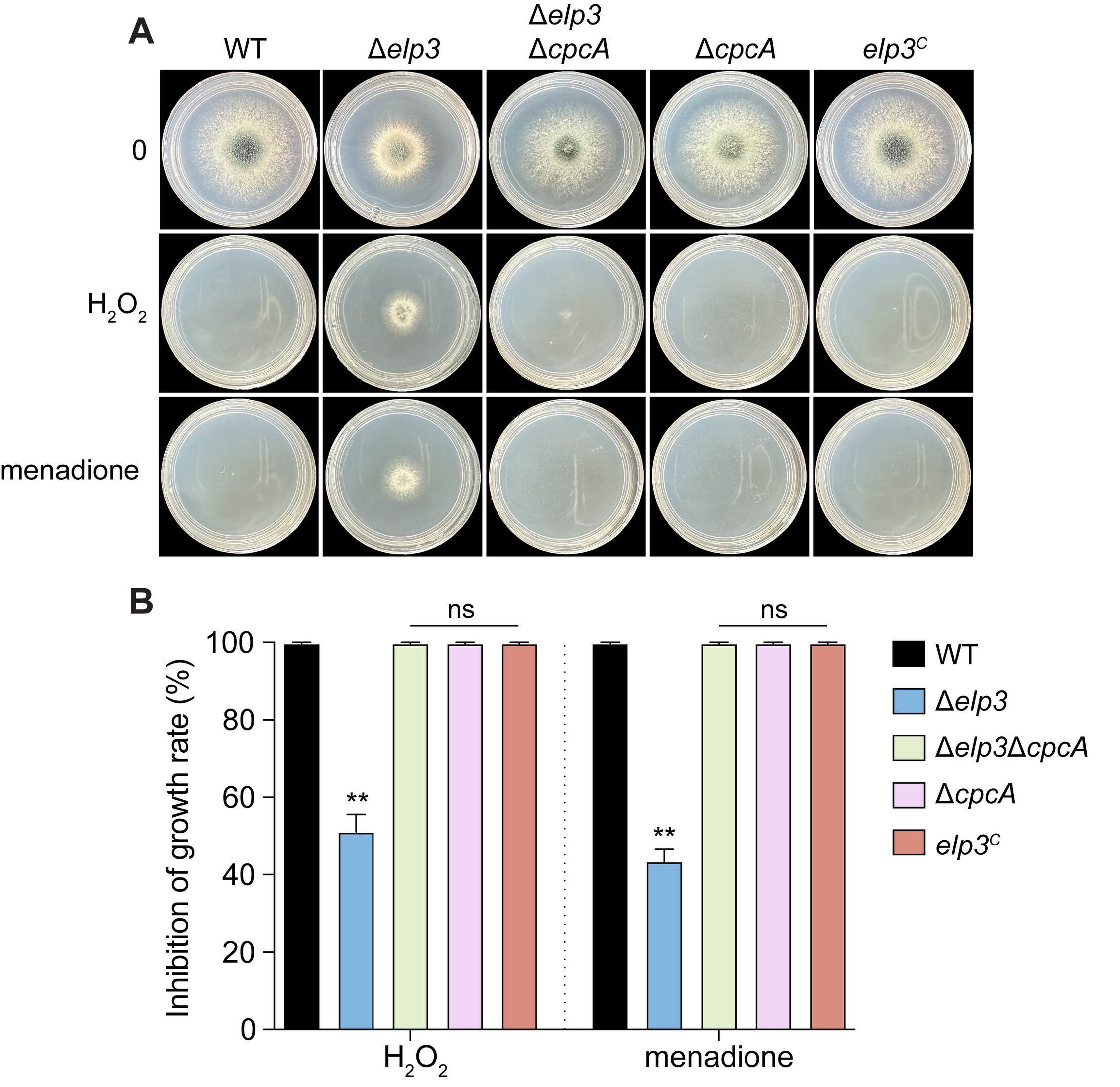

Supplement: S10 Fig — (A) Colony morphology of the wild-type, Δelp3, Δelp3ΔcpcA, ΔcpcA and complementation strains grown on solid MM in the presence of 4 mM H2O2 and 10 μM menadione at 37°C for 48 h. (B) Relative hyphal growth inhibition of the indicated strains at 37°C for 48 h. The data are presented as the mean ± SEM (standard error of the mean) of three independent experiments. Statistical analysis was performed using one-tailed, unpaired t tests. **p < 0.01; ns, not significant. (TIF) [file ppat.1010976.s010.tif]

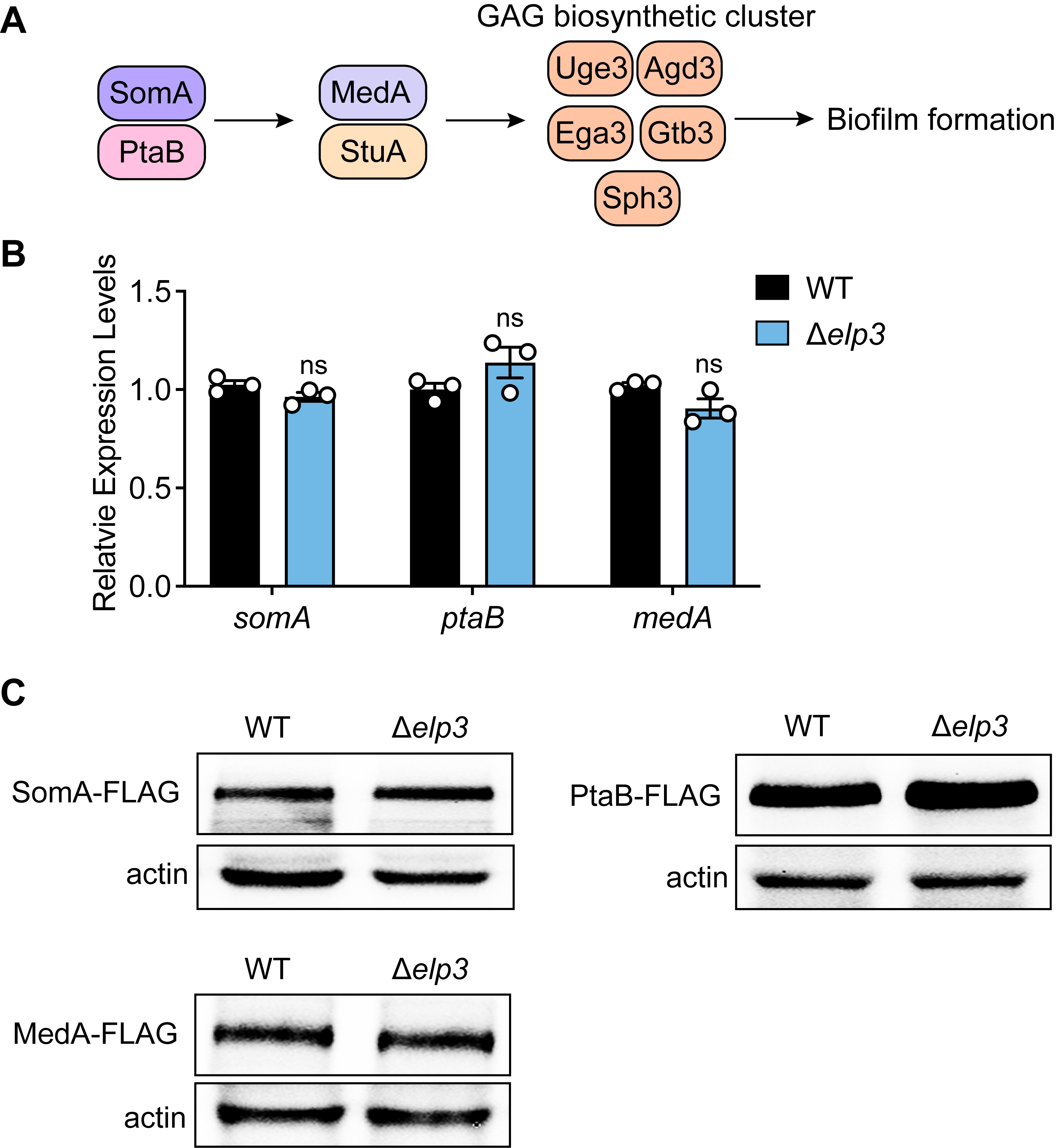

Supplement: S11 Fig — (A) Schematic diagram of GAG biosynthesis pathway. (B) Quantitative real-time RT-PCR analysis of somA, ptaB and medA in the wild-type and Δelp3 strains. The mRNA levels were normalized to the reference gene tubA. The data are presented as the mean ± SEM (standard error of the mean) of three independent experiments. Statistical analysis was performed using one-tailed, unpaired t tests. ns, not significant. (C) Western blots show the protein expression of SomA, PtaB and MedA in the wild-type and Δelp3 strains. β-actin served as the loading control. (TIF) [file ppat.1010976.s011.tif]

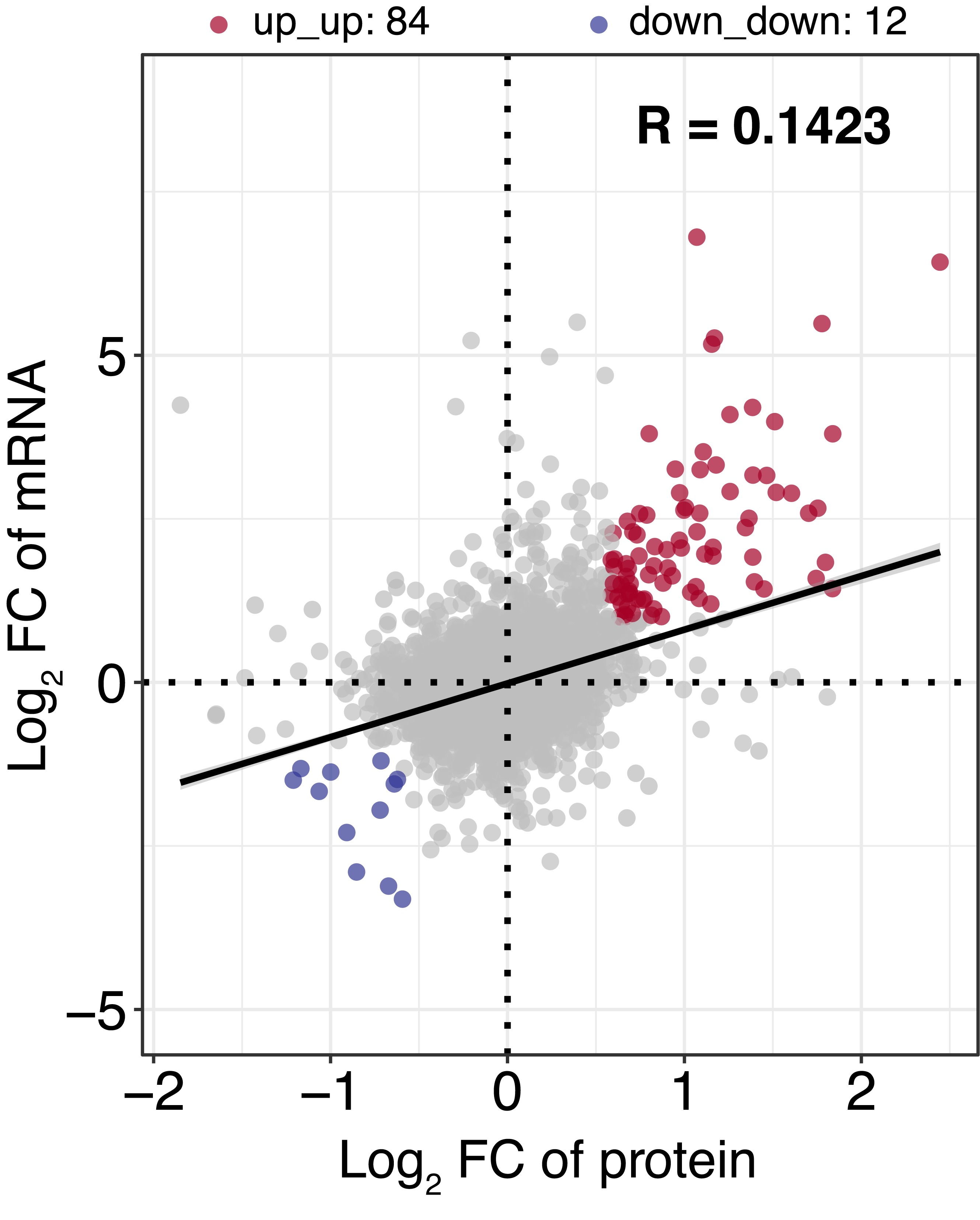

Supplement: S12 Fig — Common significant regulation at mRNA and protein levels is indicated in red (upregulation) and blue (downregulation), respectively. R, Pearson correlation coefficient. (TIF) [file ppat.1010976.s012.tif]
