# Supplementary material for: Aspergillus fumigatus Elongator complex subunit 3 affects hyphal growth, adhesion and virulence through wobble uridine tRNA modification
Source: PLoS Pathog. 2022 Nov 14;18(11):e1010976. doi: 10.1371/journal.ppat.1010976 (PMC9704764; doi:10.1371/journal.ppat.1010976)
Supplement: S1 Table — (DOCX) [file ppat.1010976.s013.docx]

**S1 Table. Strains used in this study.**

| **Strain** | **Genotype** | **Source** |
| --- | --- | --- |
| A1160 | Δ*ku80*, *pyrG* | FGSC |
| Δ*KU80* | Δ*ku80* | [[49](#_ENREF_49)] |
| Δ*elp3* | Δ*ku80*, *pyrG,* Δ*elp3*::*pyr4* | This study |
| *elp3^C^* | Δ*ku80*, *pyrG,* Δ*elp3*::*pyr4*, *elp3*::hph | This study |
| Elp3-GFP | Δ*ku80*, *pyrG*, *elp3*::GFP::*pyrG* | This study |
| Elp3-FLAG | Δ*ku80*, *pyrG*, *elp3*::FLAG::*pyr4* | This study |
| Δ*elp1* | Δ*ku80*, *pyrG,* Δ*elp1*::*pyr4* | This study |
| Δ*elp2* | Δ*ku80*, *pyrG,* Δ*elp2*::*pyr4* | This study |
| Δ*elp4* | Δ*ku80*, *pyrG,* Δ*elp4*::*pyr4* | This study |
| Δ*elp5* | Δ*ku80*, *pyrG,* Δ*elp5*::*pyr4* | This study |
| Δ*elp6* | Δ*ku80*, *pyrG,* Δ*elp6*::*pyr4* | This study |
| *elp3*^C129A^ | Δ*ku80*, *pyrG,* Δ*elp3*::*pyr4*, *elp3*^C129A^::*hph* | This study |
| *elp3*^C132A^ | Δ*ku80*, *pyrG,* Δ*elp3*::*pyr4*, *elp3*^C132A^::*hph* | This study |
| *elp3*^C129AC132A^ | Δ*ku80*, *pyrG,* Δ*elp3*::*pyr4*, *elp3*^C129AC132A^::*hph* | This study |
| *elp3*^Y551A^ | Δ*ku80*, *pyrG,* Δ*elp3*::*pyr4*, *elp3*^Y551A^::*hph* | This study |
| *elp3*^Y552A^ | Δ*ku80*, *pyrG,* Δ*elp3*::*pyr4*, *elp3*^Y552A^::*hph* | This study |
| *elp3*^Y551AY552A^ | Δ*ku80*, *pyrG,* Δ*elp3*::*pyr4*, *elp3*^Y551AY552A^::*hph* | This study |
| *elp3*^T125A^ | Δ*ku80*, *pyrG,* Δ*elp3*::*pyr4*, *elp3*^T125A^::*hph* | This study |
| *elp3*^R549A^ | Δ*ku80*, *pyrG,* Δ*elp3*::*pyr4*, *elp3*^R549A^::*hph* | This study |
| Δ*elp3 OE::uge3* | Δ*ku80*, *pyrG,* Δ*elp3*::*pyr4, gpdA(p)::uge3::hph* | This study |
| Δ*elp3 OE::agd3* | Δ*ku80*, *pyrG,* Δ*elp3*::*pyr4, gpdA(p)::adg3::hph* | This study |
| Δ*elp3 tQ^UUG^* | Δ*ku80*, *pyrG,* Δ*elp3*::*pyr4*, *tQ^UUG^*::*hph* | This study |
| Δ*elp3 tK^UUU^* | Δ*ku80*, *pyrG,* Δ*elp3*::*pyr4*, *tK^UUU^*::*hph* | This study |
| Δ*elp3 tE^UUC^* | Δ*ku80*, *pyrG,* Δ*elp3*::*pyr4*, *tE^UUC^*::*hph* | This study |
| Δ*elp3 tQ^UUG^ tK^UUU^* | Δ*ku80*, *pyrG,* Δ*elp3*::*pyr4*, *tQ^UUG^ tK^UUU^*::*hph* | This study |
| Δ*elp3 tE^UUC^ tK^UUU^* | Δ*ku80*, *pyrG,* Δ*elp3*::*pyr4*, *tE^UUC^ tK^UUU^*::*hph* | This study |
| Δ*elp3 tQ^UUG^ tE^UUC^* | Δ*ku80*, *pyrG,* Δ*elp3*::*pyr4*, *tQ^UUG^ tE^UUC^*::*hph* | This study |
| Δ*elp3 tQ^UUG^ tK^UUU^ tE^UUC^* | Δ*ku80*, *pyrG,* Δ*elp3*::*pyr4*, *tQ^UUG^ tK^UUU^ tE^UUC^*::*hph* | This study |
| Δ*cpcA* | Δ*ku80*, *pyrG,* Δ*cpcA*::*hph* | This study |
| Δ*elp3*Δ*cpcA* | Δ*ku80*, *pyrG,* Δ*cpcA*::*hph,* Δ*elp3*::*pyr4* | This study |
| H3K14R | Δ*ku80*, *pyrG,* K14R::*pyr4* | This study |
| SomA-FLAG | Δ*ku80*, *pyrG*, *somA*::FLAG::*pyr4* | This study |
| Δ*elp3* SomA-FLAG | Δ*ku80*, *pyrG,* Δ*elp3*::*pyr4*, *somA*::FLAG::*hph* | This study |
| PtaB-FLAG | Δ*ku80*, *pyrG*, *ptaB*::FLAG::*pyr4* | This study |
| Δ*elp3* PtaB-FLAG | Δ*ku80*, *pyrG,* Δ*elp3*::*pyr4*, *ptaB*::FLAG::*hph* | This study |
| MedA-FLAG | Δ*ku80*, *pyrG*, *medA*::FLAG::*pyr4* | This study |
| Δ*elp3* MedA-FLAG | Δ*ku80*, *pyrG,* Δ*elp3*::*pyr4*, *medA*::FLAG::*hph* | This study |
