# Supplementary material for: Aspergillus fumigatus Elongator complex subunit 3 affects hyphal growth, adhesion and virulence through wobble uridine tRNA modification
Source: PLoS Pathog. 2022 Nov 14;18(11):e1010976. doi: 10.1371/journal.ppat.1010976 (PMC9704764; doi:10.1371/journal.ppat.1010976)
Supplement: S2 Table — (DOCX) [file ppat.1010976.s014.docx]

**S2 Table. Primers used in this study.**

| **Primer** | **Sequence 5'-3'** |
| --- | --- |
| Elp3-P1 | tggttgacagcctcgtatgt |
| Elp3-P2 | cgatgataccggggcaattg |
| Elp3-P3 | CGATTAAGTTGGGTAACGCCAggcaggtttgttatcggcaa |
| Elp3-P4 | ATAAGTAGCCAGTTCCCGAAAGCgggtgttttcaggcgtttct |
| Elp3-P5 | gtcattgtcccactcccaga |
| Elp3-P6 | caaatgcggctaggtggatc |
| Elp3-self-F | CCTCCCTTGACGGCTATCAT |
| Elp3-self-R | AGGGGCATGGGAATATCTCG |
| Elp3-F | cttacgttccctgctttccg |
| Elp3-R | CGGCGGATTTTAGGCTCAAGtctcttccgattgctcacga |
| pyr4 F | TGGCGTTACCCAACTTAATCG |
| pyr4 R | GCTTTCGGGAACTGGCTACTTAT |
| hph-F | CTTGAGCCTAAAATCCGCCG |
| hph-R | CGGAGCATTCACTAGGCAAC |
| GFP+pyrG-F | GGAGCTGGTGCAGGCGCTGG |
| GFP+pyrG-R | CTGTCTGAGAGGAGGCACTGATG |
| Elp3-GFP-P1 | GCTGCTGTGCCGGAACAATA |
| Elp3-GFP-P2 | TTTATGTCTCTTCCGCCGGA |
| Elp3-GFP-P3 | CCAGCGCCTGCACCAGCTCCTTCGTCTTCAATTGGATCGAGC |
| Elp3-GFP-P4 | CATCAGTGCCTCCTCTCAGACAGTAAttctaactcggataagcacg |
| Elp3-GFP-P5 | gtcattgtcccactcccaga |
| Elp3-GFP-P6 | caaatgcggctaggtggatc |
| Elp3-FLAG-P1 | GCTGCTGTGCCGGAACAATA |
| Elp3-FLAG-P2 | TTTATGTCTCTTCCGCCGGA |
| Elp3-FLAG-P3 | CATTCCCGGGGATCCCTCGAGTTCGTCTTCAATTGGATCGAGC |
| Elp3-FLAG-P4 | ATAAGTAGCCAGTTCCCGAAAGCTAAttctaactcggataagcacg |
| Elp3-FLAG-P5 | gtcattgtcccactcccaga |
| Elp3-FLAG-P6 | caaatgcggctaggtggatc |
| Y551A-F | AGCgcCTATGCTCGTCTTGGGTACACACTTGAC |
| Y551A-R | AAGACGAGCATAGgcGCTGCGGACACCGACTCC |
| Y552A-F | AGCTACgcTGCTCGTCTTGGGTACACACTTGAC |
| Y552A-R | AAGACGAGCAgcGTAGCTGCGGACACCGACTCC |
| Y551AY552A-F | AGCgccgctGCTCGTCTTGGGTACACACTTGAC |
| Y551AY552A-R | AAGACGAGCagcggcGCTGCGGACACCGACTCC |
| C129A-F | CATTgcTGTTTACTGCCCTGGCGG |
| C129A-R | GGGCAGTAAACAgcAATGTTACCGGTGTAGGCAATATG |
| C132A-F | TTTGTGTTTACgcCCCTGGCGGACCCGATTC |
| C132A-R | CAGGGgcGTAAACACAAATGTTACCGGTGTAGG |
| C129AC132A-F | ATTgctgtttacgcCCCTGGCGGACCCGATTC |
| C129AC132A-R | GGGgcgtaaacagcAATGTTACCGGTGTAGGCAATATG |
| T125A-F | ATTGCCTACgCCGGTAACATTTGTGTTTACTGCC |
| T125A-R | TTACCGGcGTAGGCAATATGCGGACAACG |
| R549A-F | GGTGTCgcCAGCTACTATGCTCGTCTTGGGT |
| R549A-R | ATAGTAGCTGgcGACACCGACTCCCGAGATGA |
| Elp1-P1 | caggatgcgcttgaagagtc |
| Elp1-P2 | ctttgcggcttgtagacctg |
| Elp1-P3 | CGATTAAGTTGGGTAACGCCAcatgtatcaccgaggcttgc |
| Elp1-P4 | ATAAGTAGCCAGTTCCCGAAAGCttgggccactatctgctgaa |
| Elp1-P5 | tgccaaccaattcctccctt |
| Elp1-P6 | acagccaatccaagacgaga |
| Elp1-self-F | TAGAGAAGGCGCTTTGGACA |
| Elp1-self-R | CTAAGACCAGAGCTCGAGGG |
| Elp2-P1 | cgcgggagaaagactgattg |
| Elp2-P2 | ccctggtgtttgcgagaaaa |
| Elp2-P3 | CGATTAAGTTGGGTAACGCCAgcctggcaatgatcgacaat |
| Elp2-P4 | ATAAGTAGCCAGTTCCCGAAAGCtgggtgttgaaaagctcgag |
| Elp2-P5 | tcagctactccgagaaccag |
| Elp2-P6 | cggagggttgtttcgttacg |
| Elp2-self-F | TGGCGCAGATAACAATGTGG |
| Elp2-self-R | GGTTAGACAGGCCGAGAACT |
| Elp4-P1 | acactctatgcagcccactt |
| Elp4-P2 | taccctggaatgctggacaa |
| Elp4-P3 | CGATTAAGTTGGGTAACGCCAttgagaatttgttgcggggc |
| Elp4-P4 | ATAAGTAGCCAGTTCCCGAAAGCgttcggacatagaggagcca |
| Elp4-P5 | agatgggcaaacaaggggta |
| Elp4-P6 | gcaatccagcctcacattcc |
| Elp5-P1 | catcagcgcctccatttcat |
| Elp5-P2 | tacgcctttcagctcgatct |
| Elp5-P3 | CGATTAAGTTGGGTAACGCCAtcgagcgaccggagaaatta |
| Elp5-P4 | ATAAGTAGCCAGTTCCCGAAAGCtgcgaactcacttctggttg |
| Elp5-P5 | ggtttccgcttcagtttggt |
| Elp5-P6 | atttcccctgttccctctcg |
| Elp5-self-F | ACACAGCTTCCCCACTTACA |
| Elp5-self-R | ACGACTCCTTCTCTCTCCCT |
| Elp6-P1 | tcaaacccatccgcttgttg |
| Elp6-P2 | ctcatcacactgggagcaga |
| Elp6-P3 | CGATTAAGTTGGGTAACGCCAgcgcaatcagaactggtagc |
| Elp6-P4 | ATAAGTAGCCAGTTCCCGAAAGCcagttgttgatgggaagccg |
| Elp6-P5 | tgcataaagtggcgtcgatg |
| Elp6-P6 | ccgaggatttgtcagatcgc |
| Elp6-self-F | GCAGTACCACCCCTCCTATC |
| Elp6-self-R | ACAGCACCTCTTTCTCCTCC |
| CpcA-P1 | gtgcgagctgatagagtttgg |
| CpcA-P2 | gctggcttgcgtggattc |
| CpcA-P3 | CGGCGGATTTTAGGCTCAAGgatgcggcggtgatacaac |
| CpcA-P4 | GTTGCCTAGTGAATGCTCCGctgcgcttgtgatgtcttgt |
| CpcA-P5 | aggctatgctcgacacgg |
| CpcA-P6 | aacgttgctttcgagaaccc |
| CpcA-self-F | GACCTCAGCACTCCTTCCTT |
| CpcA-self-R | GTACTGTTCACGCTGCTGAG |
| CpcA-FLAG-P1 | GTTATTGTCGATTGAATGCG |
| CpcA-FLAG-P2 | ctgccttctaagctcctccc |
| CpcA-FLAG-P3 | CATTCCCGGGGATCCCTCGAGACCCCTGTTCTGGGCAAG |
| CpcA-FLAG-P4 | GCTCCTCTTCTTTACTCTGATGAACGATGATCGATTGGTT |
| CpcA-FLAG-P5 | aggctatgctcgacacgg |
| CpcA-FLAG-P6 | GTGTCCCATGACATACTTCCC |
| SomA-FLAG-P1 | GGTAACCATGCTCTCCAGGAC |
| SomA-FLAG-P2 | CCTGATGATGGCCCGTCAAGAG |
| SomA-FLAG-P3 | CATTCCCGGGGATCCCTCGATAAGCCATCTCCGGCGCCGGTCTCAA |
| SomA-FLAG-P4 | ATAAGTAGCCAGTTCCCGAAAGCTAAATGAGTCCGTCGGTTC |
| SomA-FLAG-P5 | GTGCATCGAGGACGAAGTTG |
| SomA-FLAG-P6 | GCAGTAGTCGCTCAGAGAAC |
| MedA-FLAG-P1 | AGTATGCCCTCTCCGTTGAC |
| MedA-FLAG-P2 | TCAAGGAGGCTCTGTCAACA |
| MedA-FLAG-P3 | CATTCCCGGGGATCCCTCGAGACACCCGTGGGAAGGGTC |
| MedA-FLAG-P4 | GCTCCTCTTCTTTACTCTGATGATTCACCTTGTCTCGCAT |
| MedA-FLAG-P5 | tgatgtacgctacgagtgct |
| MedA-FLAG-P6 | cgatgggatctgtgaaggga |
| PtaB-FLAG-P1 | CAGTCAACCTCAGCAGCAG |
| PtaB-FLAG-P2 | aacttgataacagAGCCGCG |
| PtaB-FLAG-P3 | CATTCCCGGGGATCCCTCGAGTGCAGTACCCTTCTGTCTTT |
| PtaB-FLAG-P4 | GCTCCTCTTCTTTACTCTGATAATTTTAGAGATGGTTTCCG |
| PtaB-FLAG-P5 | atagtaaaggcgcggtccc |
| PtaB-FLAG-P6 | GAGAAACTCGGCGTCTTC |
| GPE-HPH-F | CCAATGGACCAGAACTACCTG |
| GPE-HPH-R | CCACAACATTAGTCAACTCCGT |
| OE-uge3-F | GGGCTGCAGGAATTCGATATCATCAATGGACAGCTACCAGCAA |
| OE-uge3-R | GGTATCGATAAGCTTGATATCCCGCTCGATATTACGACCTGA |
| OE-agd3-F | GGGCTGCAGGAATTCGATATCCTGACACTGCAATGGTCTTCG |
| OE-agd3-R | GGTATCGATAAGCTTGATATCCCTGACAGCGGATGTTACAAA |
| RT-tub-F | TTCCGTCCCGACAACTTCGT |
| RT-tub-R | TCACAGCCTTCAGCCTCACG |
| RT-medA-F | CGGCAAGACCAGCTAATCCG |
| RT-medA-R | ATTGCGAGTGCCCTAACCCA |
| RT-ptaB-F | AACGGGACACTTATCGCTCAC |
| RT-ptaB-R | ATAGACTCGGGCAAAGACGGT |
| RT-somA-F | AACCGGTAATGCCCAGACAGAT |
| RT-somA-R | GTGTCCGTTCATGTCCATGTCA |
| RT-uge3-F | GCTGTTAGCCTCCCAGTACC |
| RT-uge3-R | GGACTTGGTCGTACCCCAT |
| RT-agd3-F | ACGCGGACGTCTTCAAGGAG |
| RT-agd3-R | GTTGTGCAGACCGGTGATGG |
| RT-gtb3-F | CACTCCTCCGTGGACTGCTT |
| RT-gtb3-R | ACGGATGAAACCGCCTTGGA |
| RT-ega3-F | ACGACAAGTCCACCATCGCA |
| RT-ega3-R | GCCCAGATCCGAGTCCTTGA |
| RT-sph3-F | AGAATGTGCGGCTGCTAGGC |
| RT-sph3-R | CAGCTAGAGCCGGGTTGGAA |
| RT-CpcA-F | CCCCAACATCGCTCAAGATA |
| RT-CpcA-R | GCCACAGAGTCCTTGACG |
